# Globally consistent response of plant microbiome diversity across hosts and continents to soil nutrients and herbivores

**DOI:** 10.1038/s41467-023-39179-w

**Published:** 2023-06-14

**Authors:** Eric W. Seabloom, Maria C. Caldeira, Kendi F. Davies, Linda Kinkel, Johannes M. H. Knops, Kimberly J. Komatsu, Andrew S. MacDougall, Georgiana May, Michael Millican, Joslin L. Moore, Luis I. Perez, Anita J. Porath-Krause, Sally A. Power, Suzanne M. Prober, Anita C. Risch, Carly Stevens, Elizabeth T. Borer

**Affiliations:** 1 Department of Ecology, Evolution, and Behavior, University of Minnesota, St. Paul, MN 55108 USA; 2 Forest Research Centre, Associate Laboratory TERRA, School of Agriculture, University of Lisbon, Lisbon, Portugal; 3 Department of Ecology and Evolutionary Biology, University of Colorado, Boulder, CO 80305 USA; 4 Department of Plant Pathology, University of Minnesota, St. Paul, MN 55108 USA; 5 Health and Environmental Sciences Department, Xi’an Jiaotong-Liverpool University, Suzhou, China; 6 Smithsonian Environmental Research Center, Edgewater, MD 21037 USA; 7 Department of Integrative Biology, University of Guelph, Guelph, ON Canada; 8 Arthur Rylah Institute for Environmental Research, 123 Brown Street, Heidelberg, VIC 3084 Australia; 9 School of Biological Sciences, Monash University, 25 Rainforest Walk, Clayton, VIC 3800 Australia; 10 School of Agriculture, Food and Ecosystem Sciences, The University of Melbourne, VIC, 3010 Australia; 11 IFEVA-Facultad de Agronomía (UBA)/CONICET, Departamento de Recursos Naturales, Catedra ´ de Ecología, Av. San Martín, 4453, Buenos Aires, C1417DSE Argentina; 12 Hawkesbury Institute for the Environment, Locked Bag 1797, Penrith, NSW 2751 Australia; 13 CSIRO Environment, GPO Box 1700, Canberra, ACT 2601 Australia; 14 Swiss Federal Institute for Forest, Snow and Landscape Research, Birmensdorf, Switzerland; 15 Lancaster Environment Centre, Lancaster University, Lancaster, LA1 4YQ UK

**Keywords:** Community ecology, Grassland ecology, Microbial ecology

## Abstract

All multicellular organisms host a diverse microbiome composed of microbial pathogens, mutualists, and commensals, and changes in microbiome diversity or composition can alter host fitness and function. Nonetheless, we lack a general understanding of the drivers of microbiome diversity, in part because it is regulated by concurrent processes spanning scales from global to local. Global-scale environmental gradients can determine variation in microbiome diversity among sites, however an individual host’s microbiome also may reflect its local micro-environment. We fill this knowledge gap by experimentally manipulating two potential mediators of plant microbiome diversity (soil nutrient supply and herbivore density) at 23 grassland sites spanning global-scale gradients in soil nutrients, climate, and plant biomass. Here we show that leaf-scale microbiome diversity in unmanipulated plots depended on the total microbiome diversity at each site, which was highest at sites with high soil nutrients and plant biomass. We also found that experimentally adding soil nutrients and excluding herbivores produced concordant results across sites, increasing microbiome diversity by increasing plant biomass, which created a shaded microclimate. This demonstration of consistent responses of microbiome diversity across a wide range of host species and environmental conditions suggests the possibility of a general, predictive understanding of microbiome diversity.

## Abstract (web summary)

Drivers responsible for plant microbiome variation between sites remain elusive. Here, the authors test how soil nutrient addition and herbivore exclusion affect plant microbiota in grasslands worldwide, showing that microbiota diversity responded to environmental variation similarly across sites.

## Introduction

The pathogens, mutualists, and commensals that comprise the microbiome of all free-living organisms form some of the most diverse communities known^1–6^, and the composition and diversity of each host’s microbiome can affect host fitness and interactions with the biotic and abiotic environment^3,7–13^. Despite the importance of the microbiome to the fitness of all organisms, we lack a general understanding of the factors determining microbiome diversity or even if general principles exist. Ecological theory (i.e., Island Biogeography and Metacommunity Theory), largely developed for free-living organisms, suggests that the diversity of a community will reflect both local-scale interactions (e.g., competition for limiting resources or consumption by enemies) and larger-scale conditions that determine the diversity of the pool of species available to colonize the local community (i.e., the metacommunity)^14–17^. However, measuring the factors affecting microbiome diversity presents significant scientific challenges, because the diversity within an individual host depends on both its local and biogeographic context^18–20^. While observational studies can identify potential predictors of microbiome diversity across large, biogeographic gradients, these predictors often covary (e.g., climate, host resource supply, herbivory rates, or host abundance), obscuring mechanistic, causal relationships. Experimental manipulations of these potential drivers may reveal causal relationships at local scales^21–23^, but experiments are rarely replicated across globally relevant environmental gradients. Thus, insights from patterns observed across larger-scale gradients remain disconnected from experimental studies at single sites or in the laboratory.

Studies of plant endophytes and pathogens, critical microbiome constituents that affect plant health and production, are broadly concordant with theoretical predictions; microbial taxa are regulated by interactions of both large-scale and local factors. At large scales, the diversity of plant pathogens and commensal endophytes has been documented to vary along regional or continental scale environmental gradients in climate and host nutrient supply^6,12,24–28^, and the diversity of microbes at these large spatial scales often can predict microbiome diversity within a single host^29,30^ (but see ref. ^24^). At more local scales (e.g., within individual hosts or leaves), micro-environmental conditions can alter pathogenic and other microbial plant symbionts. For example, the abundance and diversity of fungal plant symbionts is often higher in shaded conditions^31–35^, perhaps because light limitation reduces plant defenses^31,36–38^, increases tissue nitrogen (N)^39,40^, or increases moisture and humidity^41^. Analogous to the observed large-scale gradients, variation in local conditions may control fine-scale variation in microbial colonization, survival, proliferation, and ultimately microbiome diversity^22,31–35,42^.

The importance of nutrient supply and microclimate on plant microbiomes demonstrates the need for a multi-scale approach to understanding the microbiome while also suggesting the possibility that human activities might alter microbiome diversity by increasing supplies of biologically limiting nutrients or altering herbivore density^43–45^. For example, fossil fuel combustion and agricultural fertilizer use have increased supplies of biologically limiting nutrients to Earth’s ecosystems^44,46,47^. Humans have concurrently altered the density and types of herbivores through hunting and domestic grazing^43–45^. These global-scale alterations of nutrient supplies and herbivore density changes have the potential to alter plant microbiome diversity, because increased soil nutrient supplies and reduced herbivore density can increase shading due to increased plant biomass^48^, which may increase microbiome diversity. Herbivores also may change microbiome diversity by creating wounds, directly serving as vectors^49^, or up- or down-regulating plant immune systems^50^. Increased nutrient supplies also may increase plant tissue nutrient concentrations^51^, which can alter the diversity or composition of plant microbiomes^27,52^. Increased nutrient supplies also may act on plant microbiomes by altering the plant community. For example, increased nutrient supplies often reduce plant diversity in grasslands^48,53^, which may reduce microbiome diversity if a less diverse plant community reduces the diversity of potential microbial colonists (i.e., mass effects in metacommunity models)^18,19^. Conversely, plant diversity loss may increase the abundance of generalist microbes by increasing relative host frequency (i.e., the dilution effect)^32,54–57^. Taken together, these results suggest that ongoing anthropogenic alteration of nutrient supplies and herbivore density may have complex, multi-scale effects on plant microbiome diversity that are mediated through changes in plant biomass, shading and microclimate, plant diversity, and host abundance.

Here, we determine the factors affecting prokaryotic (bacterial and archaeal) and fungal microbiome diversity, from global to micro-environmental scales, in the dominant grass hosts at 23 grassland sites in seven countries on four continents. This approach provides unique insights into the general principles operating across a wide range of environmental conditions and host species. We start by examining the environmental covariates associated with microbiome diversity estimated at two scales: (1) diversity within a single leaf (leaf-scale) and (2) total diversity summed across all samples collected in the focal host species at a site (host-population scale). We then quantify the effects of two potential mediators of plant microbiome diversity, soil nutrients and herbivory, on leaf-scale microbiome diversity by replicating an experimental manipulation of host nutrient supply (i.e., fertilization) and herbivore density (i.e., fencing) at each site, as part of the Nutrient Network (NutNet) global experiment (Table S1)^58,59^.

Previous work within the NutNet experiment has demonstrated that the nutrient treatments increase biomass^48,60^, shading^48^, leaf tissue nutrients^51^, fungal pathogen damage^61^, and soil pathogens^62^, while also reducing plant diversity^48^. The nutrient addition treatment also has been shown to increase the abundance of grasses^63,64^, the taxonomic family of our focal hosts. The fencing treatment has been found to increase plant biomass and shading^48,60^, and to mediate the effects of nutrient supply on foliar nutrients and plant diversity^48,65^. These findings suggest that the nutrient and fencing treatments are likely to alter important ecological drivers of plant microbiomes including plant biomass, plant diversity, grass host abundance, shading, and plant tissue nutrient levels.

## Results and discussion

The sites in this study include a wide range of communities dominated by herbaceous or low-statured vegetation (e.g., alpine tundra, annual grasslands, mesic grasslands, pastures, old fields, savannas), which we hereafter refer to as grasslands. Collectively, these sites span globally relevant gradients in elevation (15–2320 m), latitude (37° S–54° N), mean annual precipitation (MAP: 246–1877 mm yr^−1^), mean annual temperature (MAT: 0–18 °C), soil nutrients (0.03–1.3% nitrogen, N, 13–234 ppm phosphorus, P), aboveground live biomass (117–813 g m^−2^), and plant richness (3–22 species m^−2^, 11–86 species site^−1^). At each site, we collected leaves from the most abundant grass species in control plots, fertilized plots (increased nutrient supply), fenced plots (reduced herbivory), and fenced and fertilized plots. This sampling included data from 18 different grass species spanning 15 genera (Table S1). We surface sterilized each leaf, then determined the diversity of microbes within the leaf using amplicon sequencing for the 16S rRNA and ITS-1 regions using 2 × 250 paired ends on an Illumina MiSeq platform. Further details are presented in “Methods”.

**Fig. 1:**
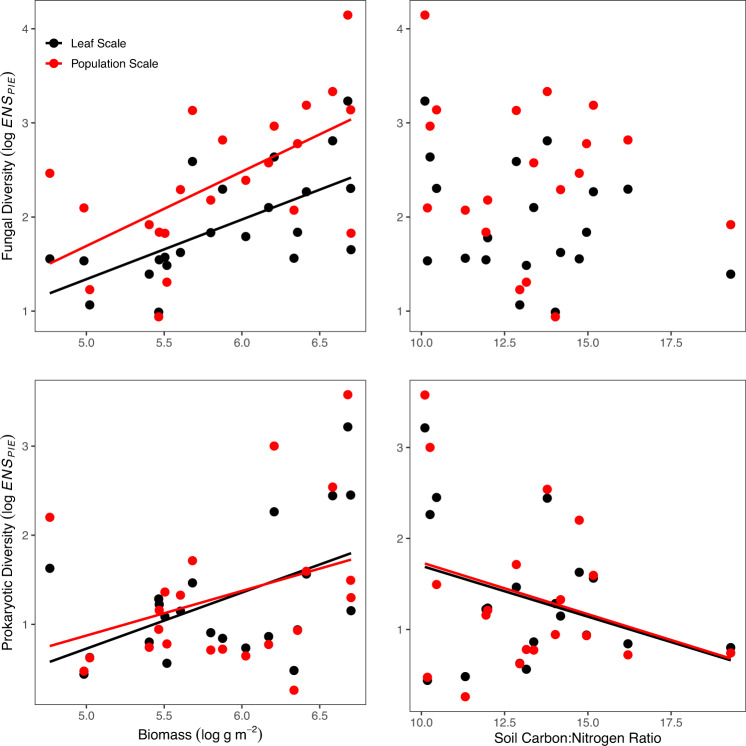
Population- and leaf-scale fungal and prokaryotic diversity are positively correlated with plant biomass (*n* = 22) and prokaryotic diversity is negatively correlated with soil carbon:nitrogen ratio (*n* = 18) based on multi-model inference from a suite of mixed effects models. Leaf-scale diversity is only from control plots. All tests are two-tailed.

In total, we acquired microbial DNA sequence data from 732 leaf samples (Table S1). Across all samples, we detected a total of 16,924 unique fungal exact sequence variants^66^ (ESVs; i.e., groups of identical sequences) and 49,905 unique prokaryotic (bacterial or archaeal) ESVs. We used these sequence data to calculate diversity at two scales: leaf scale (diversity within a single leaf sample) and host-population scale (the cumulative diversity summed across all samples of a host species collected at a site). At both scales, we measured diversity using the Effective Number of Species based on the Probability of Interspecific Encounter (*ENS*_*PIE*_) (Fig. 1). *ENS*_*PIE*_ is the estimated number of equally abundant taxa and is robust to the presence of rare species and unequal sampling intensity that typify these types of sequence data^67,68^.

### Microbiome diversity under control conditions

**Fig. 2:**
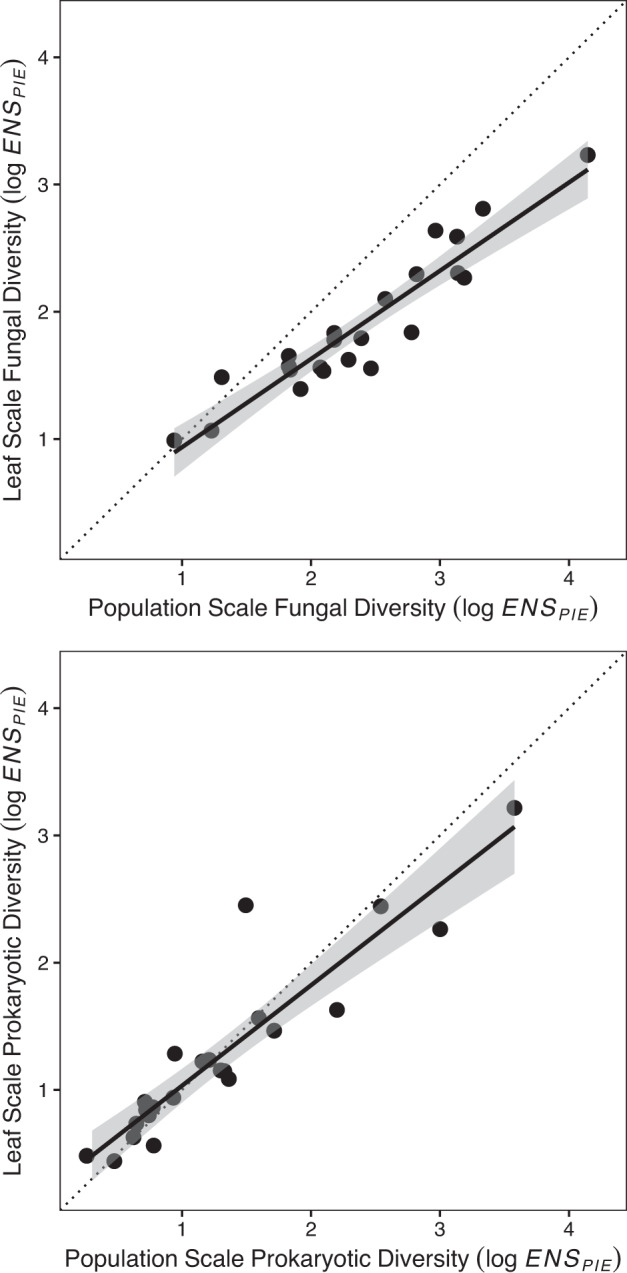
Leaf-scale fungal diversity is constrained by population-scale fungal diversity, whereas population-scale and leaf-scale bacterial diversity are similar across sites (*n* = 23). Leaf-scale diversity is only from control plots. Dotted line shows 1:1 relationship. Gray shading indicates 95% confidence envelope. All tests are two-tailed.

Sites varied widely in population-scale fungal (mean = 14.1, range = 2.6–63.2) and prokaryotic (mean = 5.9, range = 1.3–35.8) diversity in unmanipulated plots. Leaf-scale fungal (mean = 7.4, range = 2–15.7) and prokaryotic (mean= 4.7, range=1.6–25.6) diversity were similarly variable among sites (Fig. 2). Because diversity (i.e., *ENS*_*PIE*_) combines both the number and evenness of the taxa in the community, it is much lower than the raw richness, the number of ESVs, in microbial sequence samples, so comparison to other studies requires care. In samples taken from unmanipulated control plots, mean leaf-scale fungal richness was 124.6 (range = 22.7–235.9), and prokaryotic richness was 517.1 (range = 229.8–1081.6).

As predicted by community theory and observations from free-living taxa^14, 15,17,19,69^, fungal (*r* = 0.95, d.f. = 21, *p* < 0.001) and prokaryotic (*r* = 0.93, d.f. = 21, *p* < 0.001) microbiome diversity at the leaf and host-population scale were highly correlated (Fig. 2). Leaf-scale fungal diversity was less than host-population scale diversity in most cases (Fig. 2), as has been found at larger scales in biogeographical surveys of free-living plants and animals^17,69^. In contrast, prokaryotic diversity was often similar at the host-population and leaf-scales, suggesting that prokaryotic communities may be more controlled by supply of microbial colonists from larger scales (i.e., mass effects in metacommunity models) than by local competitive interactions^15–17,19,69^. The strong dependence of leaf-scale microbiome diversity on the larger spatial context (Fig. 2) highlights the relevance of a range of existing community theory to research on microbiomes^17,19^, and suggests fruitful avenues for theoretical and empirical investigation at the nexus of biogeography and microbial ecology.

Fungal and prokaryotic diversity were highly correlated at the leaf-scale across sites (*r* = 0.88, d.f. = 21, *p* < 0.001) suggesting that the same processes determine the diversity of these disparate taxonomic groups across a wide range of biotic and abiotic environments and a variety of host species. We examined the effects of potential drivers of microbiome diversity including climate (MAP, MAT, and MAP divided by potential evapotranspiration), aboveground plant biomass, plant diversity, shading, and soil chemistry (soil N, P, C:N, and pH) (Tables S2 and S3). Host-population scale fungal diversity was highest at sites with abundant live plant biomass, but did not vary consistently with soil C:N (Fig. 1; Table S2). Population-scale prokaryotic diversity showed a weak positive relationship with live biomass and also declined with ambient soil C:N suggesting that soil nutrients may limit prokaryote microbiome diversity (Fig. 1). At the leaf-scale, prokaryotic diversity was positively correlated with live plant biomass and negatively correlated with host abundance (Table S3). Taken together these results demonstrate that fungal and prokaryotic microbiome diversity is generally highest at sites with high biomass, and that prokaryotic diversity is higher at sites with higher soil N availability (low C:N) and low focal host abundance.

### Effects of nutrient addition and herbivore reduction on microbiome diversity

**Fig. 3:**
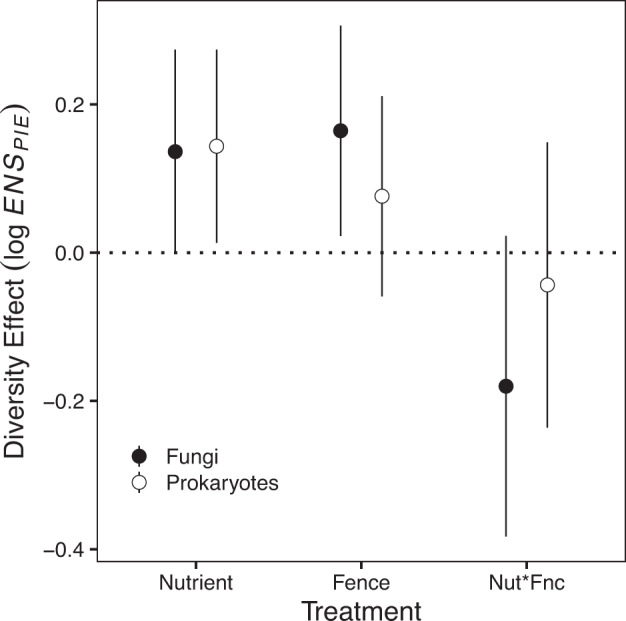
Fertilization increases leaf-scale fungal and prokaryote diversity, and fencing increases leaf-scale fungal diversity. “Nutrient” is the effect of fertilization, “Fence” is the effect of fencing, and “Nut*Fnc” is the interaction between Fertilization and Fencing with zero indicating additivity. Error bars represent the standard error of the effect estimate based on 515 observations nested by site, block, and plot in a mixed effects model (Table S4).

**Fig. 4:**
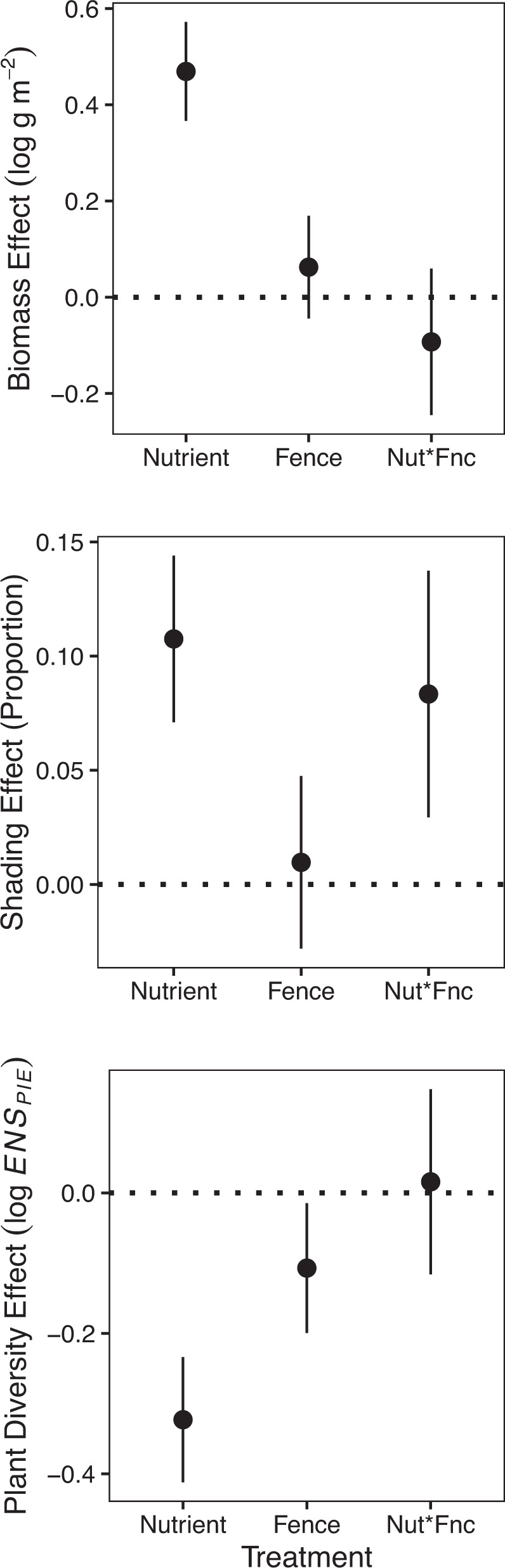
Fertilization increases biomass and shading and reduces plant diversity. Fencing reduced plant diversity and increased effects of fertilization on shading (positive interaction). “Nutrient” is the effect of fertilization, “Fence” is the effect of fencing, and “Nut*Fnc” is the interaction between Fertilization and Fencing with zero indicating additivity. Error bars represent the standard error of the effect estimate based on 515 observations nested by site, block, and plot in a mixed effects model (Table S5). All tests are two-tailed.

Given the importance of soil nutrients and plant biomass as predictors of microbiome diversity in our observational (Control Plot) data, we analyzed the effects of an experimental manipulation of soil nutrient supply and herbivore abundance replicated at all sites in the study. In addition to the direct effect on soil nutrient supply, nutrient addition and herbivore reduction treatments are important global drivers of plant host biomass^48,60^ and community composition^48,65^. Across sites, experimental nutrient addition increased fungal and prokaryotic diversity and herbivore reduction increased fungal diversity (Fig. 3; Table S4). Manipulation of nutrients and herbivore density also altered the local conditions experienced by the host plants (Fig. 4; Table S5), including those that were consistently important in the analysis of the observational data (e.g., plant biomass and host plant abundance). Fertilized plots were shadier and had more live biomass than unfertilized plots, and fertilization reduced plant diversity and focal host abundance. Plant diversity was reduced in fenced plots, and plots that were both fenced and fertilized were the shadiest (Fig. 4). These experimental results are consistent with the among-site relationships in the observational data; fungal diversity was high where plant biomass and shading were high under both observational and experimental conditions. In both the observational and experimental data, the link between prokaryotic diversity and plant biomass and shading was weaker but concordant with fungal diversity effects.

**Fig. 5:**
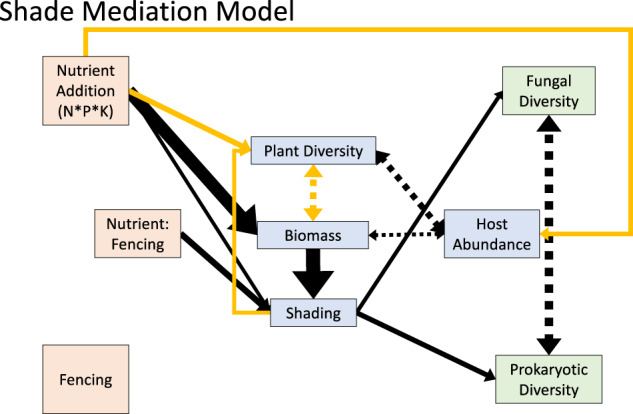
Nutrient and fencing effects on leaf-scale endophyte diversity are mediated by biomass effects on shade. Arrow width represents magnitude of standardized coefficients (Table S6). Double-headed, dashed arrows indicate relationships modeled as correlated errors. Black arrows represent positive coefficients and orange arrows represent negative coefficients. All tests are two-tailed.

Motivated by these univariate analyses, we used structural equation models (SEMs) to investigate the specific pathways by which increased nutrient supply or reduced herbivory altered leaf-scale fungal and prokaryotic microbiome diversity. We started with a full model that included direct effects of the nutrient addition and fencing treatments on leaf-scale microbiome diversity and indirect effects of the treatments mediated by changes in the local conditions that were likely to impact microbiome diversity based on existing studies and our univariate analyses: plant diversity^18, 19,32,54–57^, host abundance^18,19,54^, plant biomass^23^, and shading^31–34^. We compared this full model (Model 1; Figure S1, S2, Table S4) to two nested models that tested whether all treatment effects are mediated through changes in the plant community (plant diversity, host abundance, plant biomass, and shading) (Model 2, Figure S3; Table S4) and the most parsimonious model in which all nutrient and herbivore effects are mediated by the plant community’s impacts on ground-level light availability (i.e., shading; Model 3; Fig. 5; Table S4). The fit of these models did not differ based on likelihood ratio tests, and the most parsimonious model (Model 3) had the lowest AIC (Table S4), suggesting that a model in which all the effects of reduced herbivory and increased nutrients on microbiome diversity were mediated by changes in shading was consistent with the data (Fig. 5).

The most parsimonious model (Model 3) accounted for a large proportion of the variance in leaf-scale fungal diversity (conditional *r*^2^ = 0.49; marginal *r*^2^ = 0.12) leaf-scale prokaryotic diversity (conditional *r*^2^ = 0.57; marginal *r*^2^ = 0.01), plant biomass (conditional *r*^2^ = 0.51; marginal *r*^2^ = 0.09), shading (conditional *r*^2^ = 0.68; marginal *r*^2^ = 0.21), plant diversity (conditional *r*^2^ = 0.66; marginal *r*^2^ = 0.02), and host abundance (conditional *r*^2^ = 0.37; marginal *r*^2^ = 0.02). These structural equation models revealed that the increase in microbiome diversity was associated with increased shading that arose from the nutrient addition and fencing treatments, suggesting an important role of microclimate in shaping a host’s microbiome (Fig. 5). Although we present the most parsimonious model here, details on all models are presented in Table S6.

## Conclusions

The positive relationships between microbiome diversity and shading are consistent for both fungi and bacteria and remain consistent across a wide range of host species growing under very different site conditions, suggesting that shading is a general predictor of plant microbiome diversity. These findings align with observational studies showing the importance of microclimate and shading in forested systems. For example, foliar pathogen abundance on tree seedlings has been found to be higher in low-light and high nutrient conditions^31^, and fungal endophytes in trees are more abundant and diverse in shaded parts of the canopy^32, 33^. In addition, shading and the associated increases in humidity have been shown to affect host-pathogen interactions; many pathogens are more abundant in shaded or moist conditions^34,35,41^. These effects may reflect reduced plant defenses found in low light conditions^31,36–38^. Low light conditions also can lead to increased tissue nitrogen^39^, a potential limiting nutrient for microbiome communities^27,31,40^. These effects also may arise from higher leaf wetness in shaded conditions^41^. While we are not yet able to resolve these more proximal effects of shading, previous work within this experimental network has shown that the nutrient addition treatments can increase the plant tissue nutrient concentrations and prevalence of fungal pathogens, but nutrients do not consistently modify foliar thickness (specific leaf area)^51,61^. These results suggest that altered tissue chemistry may influence microbiome diversity; however, the inconsistent effects of nutrients (i.e., soil C:N vs nutrient addition) and the consistent effects of plant biomass suggest that biomass and shading will remain important determinants of the microbiome.

While the SEM model in which all effects of nutrients and herbivores on microbiome diversity are mediated by changes in shading (Model 3) was comparable to more complex models, this does not preclude the importance of mechanisms represented in the more complex models or effects mediated by processes we were not able to measure. There are likely a wide array of processes acting concurrently to determine microbiome composition and diversity. For example, other studies conducted in the NutNet experiment have shown that the fertilization treatments can increase both arthropod abundance and leaf damage^61,70^, which have been shown to be associated with more diverse fungal endophyte communities^49^. Other studies at these sites have shown that experimental nutrient addition alters the soil microbiome and root endophyte communities^62,71^, which may serve as innocula for the foliar microbiome^72,73^.

Differences among host species are also likely to be an important factor shaping the plant microbiome^52,73,74^. For example, a recent observational study found that variation in the fungal microbiome in tropical tree species was associated with a correlated, common suite of leaf traits (e.g., tissue nutrient concentration or specific leaf area, SLA) which represent a spectrum from slow to fast return on investments in leaf nutrients or carbon (i.e., the leaf economic spectrum)^52,75^. In the current study, we examined the dominant grass at each site, and most host species were only sampled at a single site. Nonetheless, we found that biomass was consistently associated with microbial diversity across sites, where hosts differed, and within sites, where host identity was held constant, pointing to the generality of biomass and shading in shaping the host microbiome. While this approach provides consistency among sites by sampling the host that is dominant at each site, the design does not allow a direct comparison of the effects of among host variation on the microbiome. A natural follow up to this work would be to sample a range of host species along the leaf economic spectrum within each site, and test whether these traits predict the microbiome composition or diversity within or across sites^52^. A complementary study would isolate host identity from site conditions by sampling the microbiome of a widespread species across a range of sites.

Our results demonstrate the potential for human activities, such as increasing nutrient supply rates and altering herbivore abundances, to cause global shifts in microbiome diversity^43–45^. Because the composition and diversity of the microbiome can alter host fitness and interactions with the environment^3,7–13^, understanding the local and biogeographic processes governing plant microbiome diversity has important implications for the productivity and diversity of grassland ecosystems^3,7–13^. More generally, we show striking similarity in the processes governing the diversity of microbes from different taxonomic domains across a wide range of different host species growing under ambient and experimental conditions in 23 different grasslands spanning seven countries on four continents. These results also map onto core theoretical frameworks in community ecology (e.g., metacommunity and island biogeography theory)^14–16^. Taken together, these results suggest that there may be general principles governing microbiome diversity across spatial scales and host species.

## Methods

We conducted this work using sites that are part of the Nutrient Network Experiment (NutNet; www.nutnet.org), a globally replicated experiment manipulating elemental nutrient supplies and herbivore density in grasslands worldwide^58,59^. We used amplicon sequencing to measure relative abundances of fungal (ITS1) and prokaryotic (16S) diversity in the leaves of the most widespread grass at each of 23 grassland sites. We also measured biotic and abiotic variables as described below.

Data were collected within the context of an experiment composed of a factorial combination of two treatments: nutrient addition and herbivore reduction applied to 5 × 5 m plots. The nutrient addition treatment entailed the addition of nitrogen (N), phosphorus (P), potassium (K), and micronutrients (10 g N m^−2^ yr^−1^ as timed-release urea,10 g P m^−2^ yr^−1^ as triple-super phosphate, 10 g K m^−2^ yr^−1^ as potassium sulfate, and 100 g m^−2^ yr^−1^ of a micronutrient mix; 6% Ca, 3% Mg, 12% S, 0.1% B, 1% Cu, 17% Fe, 2.5% Mn, 0.05% Mo, and 1% Zn). N, P, and K were applied annually, and the micronutrient mix was applied once at the start of the study. Herbivore reduction was accomplished by fencing plots with 230 cm tall fences to exclude nonclimbing mammals. The lower 90 cm of the fence was 1 cm wire mesh, which included an additional 30 cm outward facing flange stapled to the ground to exclude digging animals. The upper portion of the fence was composed of strands of barbless wire. Slight deviations in fence design are detailed by Borer et al.^59^. The experiment was a completely randomized block design with most sites (77%) having 3 replicate blocks (range = 2–5) and the treatments had been applied for a median of 8 years at the time of sampling (range = 2–8). Treatments were applied to 5 × 5 m plots, the experimental unit.

### DNA Extraction and Sequencing

The focal hosts included 18 grass species from 15 genera (Table S1). At peak biomass, we collected the most mature, non-senescent leaves totaling at least 250 mg of fresh tissue from each of three individuals of the focal grass species in each plot for a median of 36 leaf samples per site (range = 12–54). Samples were stored in CTAB buffer and shipped to the University of Minnesota for processing and sequencing. Upon receipt, leaves were surface sterilized by immersing them for 1 minute each successively in water, 75% ethanol, 0.4125% sodium hypochlorite (bleach solution), 75% ethanol and sterile distilled water. Following surface-sterilization, samples were stored at −80 °C. Subsequently, leaves were ground in liquid nitrogen with a mortar and pestle, and total genomic DNA was extracted using the Qiagen Plant Mini Extraction Kit (Qiagen N.V., Venlo, Netherlands), and standardized to 20 ng μl^−1^. Amplicon sequencing was performed for the v4 16 S rRNA and ITS-1 regions using 2 × 250 paired end on an Illumina MiSeq platform, according to standard protocols at the University of Minnesota Genomics Center (UMGC)^76^. Samples for v4 16S rRNA amplicon sequencing were split across 9 sequencing runs and samples for ITS-1 amplicon sequencing were split across 7 sequencing runs.

All data processing and analysis was performed in R (v. 4.1.2)^77^. Raw sequencing data were filtered, trimmed, and merged into exact sequence variants (ESVs; sequences that are identical) using the default pipeline in ‘dada2’ package (v. 1.14) for each sequencing run independently^78^. Callahan et al.^66^ and Porath-Krause, et al.^68^ provide further discussion of sequence grouping and use of ESVs. Taxonomic assignment also was performed using the ‘dada2’ package (v. 1.14). The SILVA SSU v132 database was used to assign bacterial taxa to 16S rRNA reads^79^. For fungal taxa assignments to ITS-1 reads, the UNITE database (version 8.2) was used. ESVs were filtered for chloroplast and mitochondrial contamination^80^. Processed data from each sequencing run were combined with their respective taxonomy classification into phyloseq objects using the ‘phyloseq’ package (v 1.30.0)^81^. Phyloseq objects were combined based on sample names into one final phyloseq object with ESVs summed among matching samples across all sequencing runs. We removed sequences that were identified as cyanobacteria or non-fungal eukaryotes. We also removed samples with <1000 reads (5% of total samples).

We obtained 33,500,410 fungal (ITS) and 40,609,045 bacterial and archaeal (16S) DNA sequences from the 705 leaf samples with a mean of 48,778 (range = 1019–245,463) fungal and 60,883 (range = 1110–739,738) prokaryotic (bacterial and archaeal) reads per sample. We detected 16,924 unique fungal ESVs. Of these fungal taxa, 94% had matches at the Phylum level, 76% at the Class level, and 71% at the Order level. The ESVs with taxonomic matches represented 9 phyla, 38 classes, and 115 orders (Fig. S4), with the majority (96%) being either Ascomycota (74%) or Basidiomycota (25%). We detected 49,905 unique bacterial or archaeal ESVs. Of these taxa, 24% had matches at the Domain level, 22% had matches at the Phylum level, 22% at the Class level, and 20% at the Order level. The ESVs with taxonomic matches were 99.5% bacteria and represented 42 phyla, 94 classes, and 192 orders (Fig. S4), with the majority (81%) being either Proteobacteria (46%), Bacteroidetes (15%), Firmicutes (10%), or Actinobacteria (10%). Note that all ESVs identified as Fungi, Bacteria, or Archaea (matched and unmatched to a lower taxonomic level) were used in subsequent analyses. Using these data, we calculated fungal and prokaryotic (bacterial and archaeal) diversity at the site and leaf level.

### Environmental measurements

At the same time as we collected samples for DNA sequencing, we measured physical and biological characteristics of the environment that we expected could control microbiome diversity based on the theoretical and empirical evidence discussed above (plant biomass, plant diversity, host abundance, shading, soil resources, and climate).

We measured aboveground live plant biomass in each plot by clipping all plants in two 0.1 × 1 m strips, sorting out the current year’s growth (live biomass), drying the biomass to a constant mass at 60 °C, and weighing the dried samples to the nearest 0.01 g. We calculated plant diversity and focal host abundance based on percent cover of all vascular plant species in a 1 ×1 m plot. We used these data to estimate the relative abundance of our focal host species in each plot ($\frac{{{{{{\rm{Focal}}}}}}\; {{{{{\rm{Host}}}}}}\; {{{{{\rm{Cover}}}}}}}{{{{{{\rm{Total}}}}}}\; {{{{{\rm{Plant}}}}}}\; {{{{{\rm{Cover}}}}}}}$) and plant diversity at the plot and site scale. Our metric of plant diversity was Effective Number of Species based on the Probability of Interspecific Encounter (*ENS*_*PIE*_), which is equivalent to Inverse Simpson’s Diversity. We also used *ENS*_*PIE*_ as our metric of microbial diversity, and we discuss some of the properties of this metric in the next section.

To calculate shading, we measured the amount of photosynthetically active radiation (PAR) blocked by the plant canopy. To do this we measured PAR above the plant canopy and at ground level below the plant canopy using a ceptometer, allowing us to calculate an index of shading (1 - $\frac{{{{{{\rm{PAR}}}}}}\; {{{{{\rm{at}}}}}}\; {{{{{\rm{Ground}}}}}}\; {{{{{\rm{Level}}}}}}}{{{{{{\rm{PAR}}}}}}\; {{{{{\rm{Above}}}}}}\; {{{{{\rm{Canopy}}}}}}}$). PAR measurements were made within two hours of solar noon.

We measured soil chemistry in 10 cm deep soil cores collected prior to the application of the nutrient treatments and analyzed them for soil nutrients (C, N, P, pH) using standard methods^82^. In our models, we include total N (%), extractable P (ppm), C:N, and pH.

Climate data were accessed from the WorldClim database^83^. In our analyses, we included mean annual precipitation (MAP: 249 to 1877 mm yr^−1^), mean annual temperature (MAT: −3 to 23 °C), and a water availability index (WAI) calculated as MAP divided by potential evapotranspiration (WAI = MAP/PET).

### Diversity metrics and statistical analyses

We calculated fungal and prokaryotic diversity in all individual leaf samples collected in control or treatment plots (leaf-scale diversity). We also calculated host population-scale fungal and prokaryotic diversity by summing the abundance of all ESVs collected in the focal host at each site and calculating the diversity of the summed abundances. We calculated plant community diversity at the 1 m^2^ plot scale using the plant percent cover data.

In assessing plant and fungal diversity, we use the Effective Number of Species based on the Probability of Interspecific Encounter (*ENS*_*PIE*_). *ENS*_*PIE*_ is equivalent to Inverse Simpson’s Diversity, and is a biodiversity metric that is more robust than raw or rarefied species richness to some of the challenges associated with amplicon sequencing data, including the presence or absence of rare species, skewed abundance distributions, and uneven sampling intensity (e.g., differing number of DNA reads per sample)^67^. For example, analysis of simulated sequencing data has shown that *ENS*_*PIE*_ converges more rapidly on the underlying population diversity than richness or rarefied richness, especially when taxa abundances are highly skewed as is often the case for this type of sequencing data^68^. *ENS*_*PIE*_ estimates the number of equally abundant species or ESVs in a sample and is calculated as ${1/\mathop{\sum }\limits_{i=1}^{S}p}_{i}^{2}$ where *S* is the total number of species or ESVs and *p*_*i*_ is the proportion of the community represented by species or ESV*i*^67^.

All analyses were conducted using R version 4.1.2 (2021-11-01). For univariate analyses at the plot or leaf scale, we account for nesting of samples within sites or plots by using Mixed Effects Models (MEMs) fit with the lmer function in the lme4 R library (version 1.1-27.1) with *p*-values generated using Satterthwaite’s degrees of freedom method using the lmerTest R library (version 3.1-2). Model specifications are included in Tables S1–S3, which include the random-effects structure.

In our analyses of environmental covariates (e.g., climate, plant biomass, and shading), we used a multi-model approach to model selection using the dredge and model.avg functions in the MuMIn library (version 1.43.17)^84^, because there could be multiple models with similar AIC values. Parameter importance in the multi-model approach is the sum of the Akaike weights summed across the set of models within 4 AIC_c_ units of the top model (lowest AIC_c_) in which a parameter is included. Importance ranges from 0 (parameter has no explanatory weight) to 1 (parameter is in all top models). We standardized the input variables using the arm library (version 1.12-1).

Structural Equation Models were fit using the piecewiseSEM R library (version 2.1.2)^85^. We developed our full model (Model 1; Fig. S1) based on existing evidence for likely mechanistic links between the experimental treatments (Nutrient Addition and Grazing Reduction), the host community (Plant Diversity, Plant Biomass, Host Abundance, and Shading), and microbiome diversity (Fungal Diversity and Prokaryote Diversity). In cases where a relationship between two variables was not clearly unidirectional, we specified the relationship as correlational. These cases are specified by double-headed arrows in the Fig. 5, S1, S2, and S3 and Table S6. Overall model fit was assessed with Fisher’s C statistic. This statistic indicates that the hypothesized model is consistent with the data when p is greater than a significance threshold (*p* > 0.05)^85^. We compared these three SEM models by examining the AIC and using a likelihood ratio test using the anova function in R.

Of our 23 sites, we were missing light data at 6 sites and soils data at 4 sites. In our analyses of observational data, we present analyses for the full set of sites when possible, and we note when fewer sites are included due to missing data (Table S1). The following responses and covariates were natural log transformed to normalize residuals: host cover, plant, fungal, and prokaryotic diversity, plant biomass, soil P and N.

### Reporting summary

Further information on research design is available in the Nature Portfolio Reporting Summary linked to this article.

## Supplementary information

Supplementary Information

Reporting Summary

## Data Availability

All data used in this manuscript are archived at the Environmental Data Initiative (https://environmentaldatainitiative.org/): 10.6073/pasta/c1d1074bb1dd46d1ba037f6a80d21233. The raw sequencing data have been deposited on the National Center for Biotechnology Information Sequence Read Archive (NCBI SRA) under Bioproject PRJNA944716 (https://www.ncbi.nlm.nih.gov/bioproject/PRJNA944716). For databases used in this study, SILVA database is available at https://www.arb-silva.de; UNITE database is available at https://unite.ut.ee/repository.php.
