# Supplementary material for: Globally consistent response of plant microbiome diversity across hosts and continents to soil nutrients and herbivores
Source: Nat Commun. 2023 Jun 14;14:3516. doi: 10.1038/s41467-023-39179-w (PMC10267103; doi:10.1038/s41467-023-39179-w)
Supplement: Supplementary file 1 — Supplementary Information [file 41467_2023_39179_MOESM1_ESM.pdf]

Globally Consistent Response of Plant Microbiome Diversity Across Hosts and Continents to Soil Nutrients and Herbivores

Eric W. Seabloom, Maria C. Caldeira, Kendi F. Davies, Linda Kinkel, Johannes M. H. Knops, Kimberly J. Komatsu, Andrew MacDougall, Georgiana May, Michael Millican, Joslin L. Moore, Luis I. Perez, Anita J Porath-Krause, Sally A. Power, Suzanne M. Prober, Anita C. Risch, Carly Stevens, Elizabeth T. Borer

Supplemental Material

Figure S1. Full meta-model used in SEM. Double-headed, dashed arrows indicate relationships modeled as correlated errors.

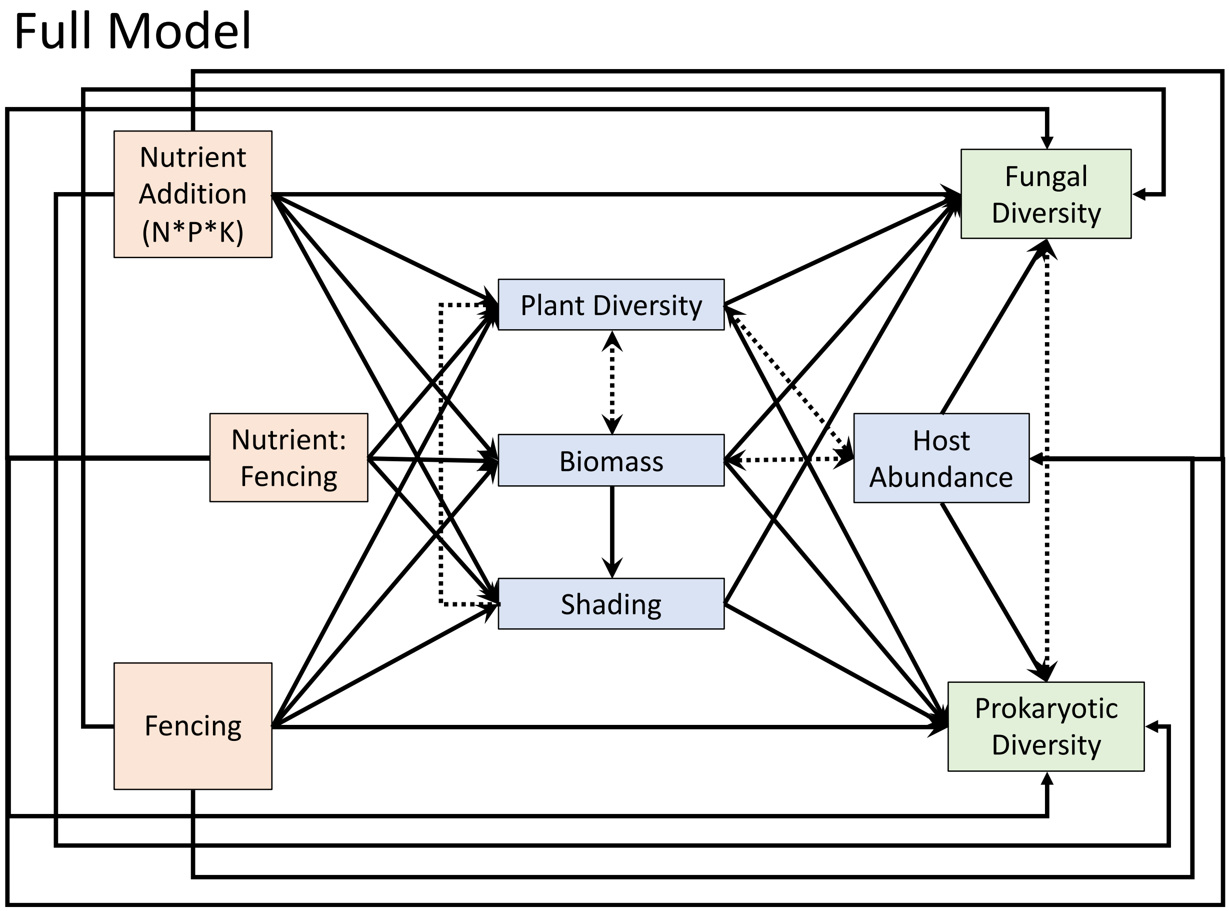

Figure S2. Full SEM (Model 1) with only significant links shown. Arrow width represents magnitude of standardized coefficients. Double-headed, dashed arrows indicate relationships modeled as correlated errors. Black arrows represent positive coefficients and orange arrows represent negative coefficients.

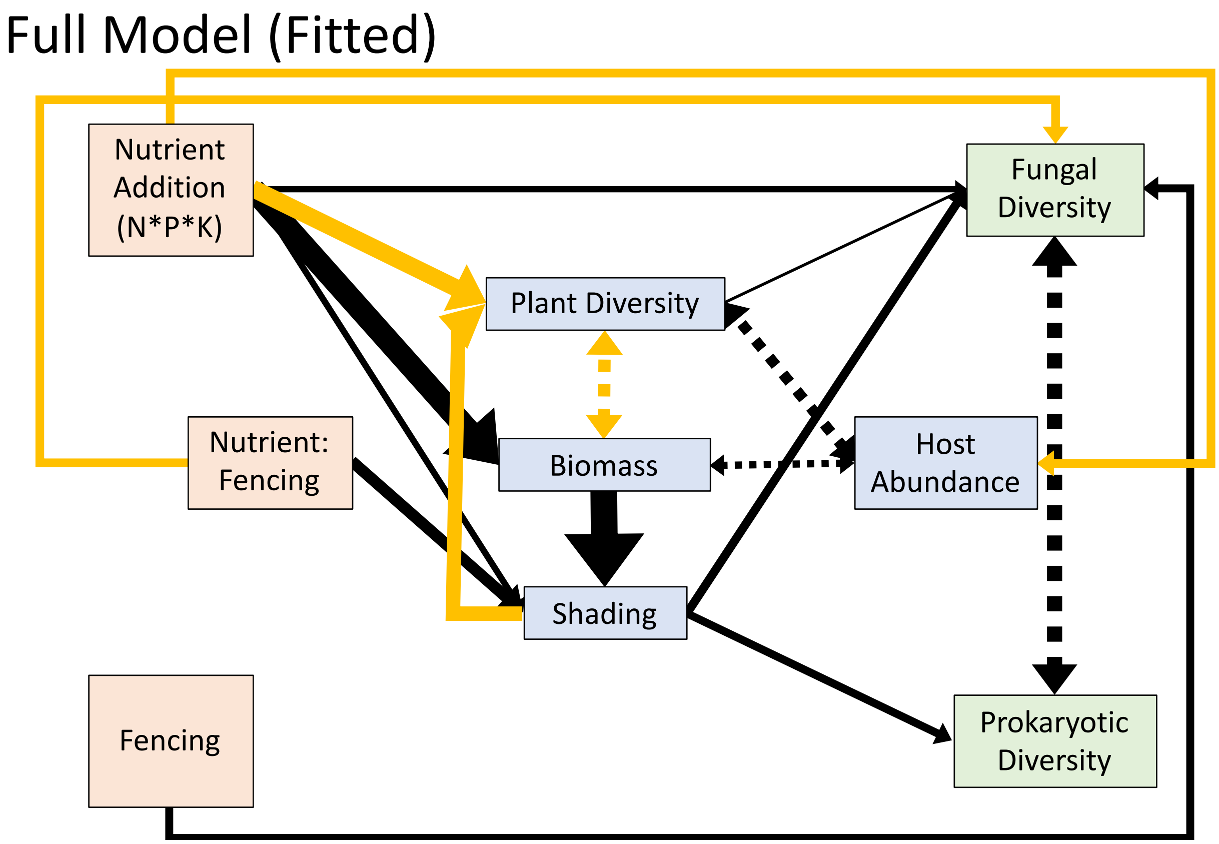

Figure S3. Plant-mediated SEM (Model 2) with only significant links shown. Arrow width represents magnitude of standardized coefficients. Double-headed, dashed arrows indicate relationships modeled as correlated errors. Black arrows represent positive coefficients and orange arrows represent negative coefficients.

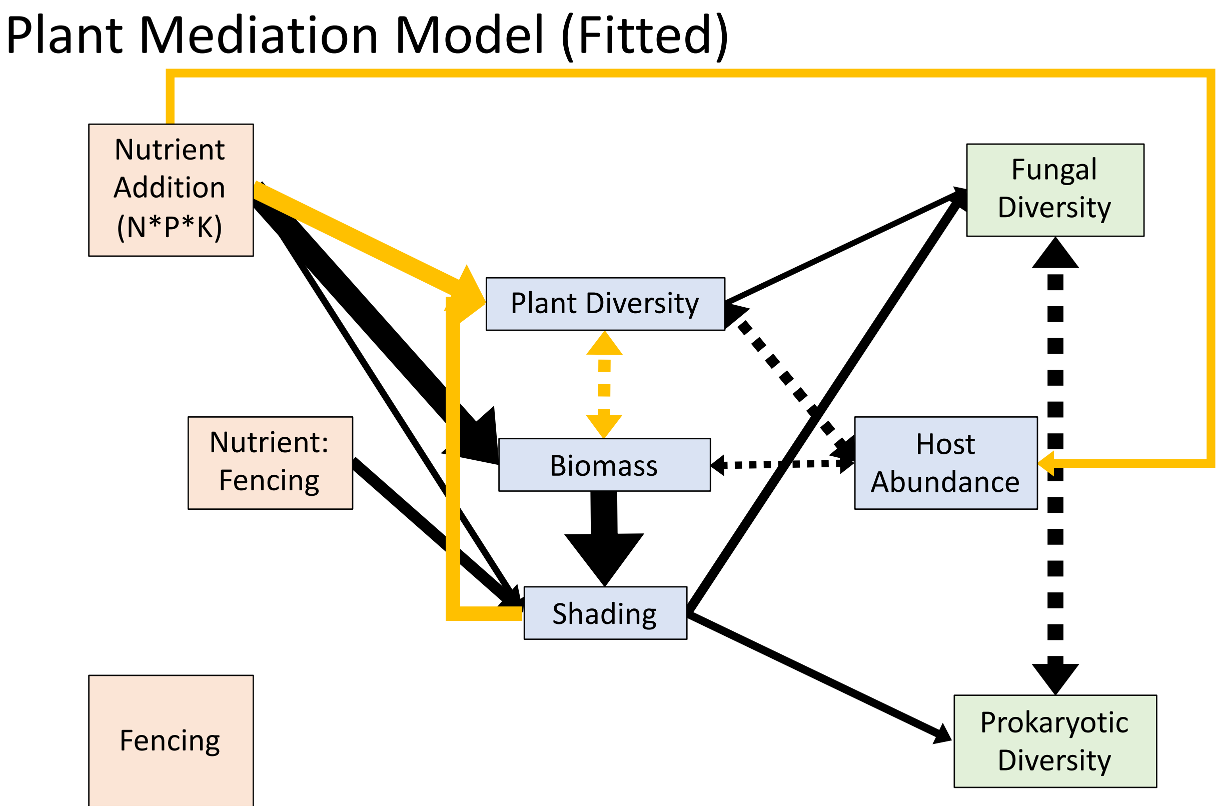

Figure S4. Relative abundance of fungal and prokaryotic (bacteria and archaea) plant symbionts. Colors represent the logged percent abundance of the fungal Class or bacterial or archaeal Phylum with sites (Panels A & B).

A.

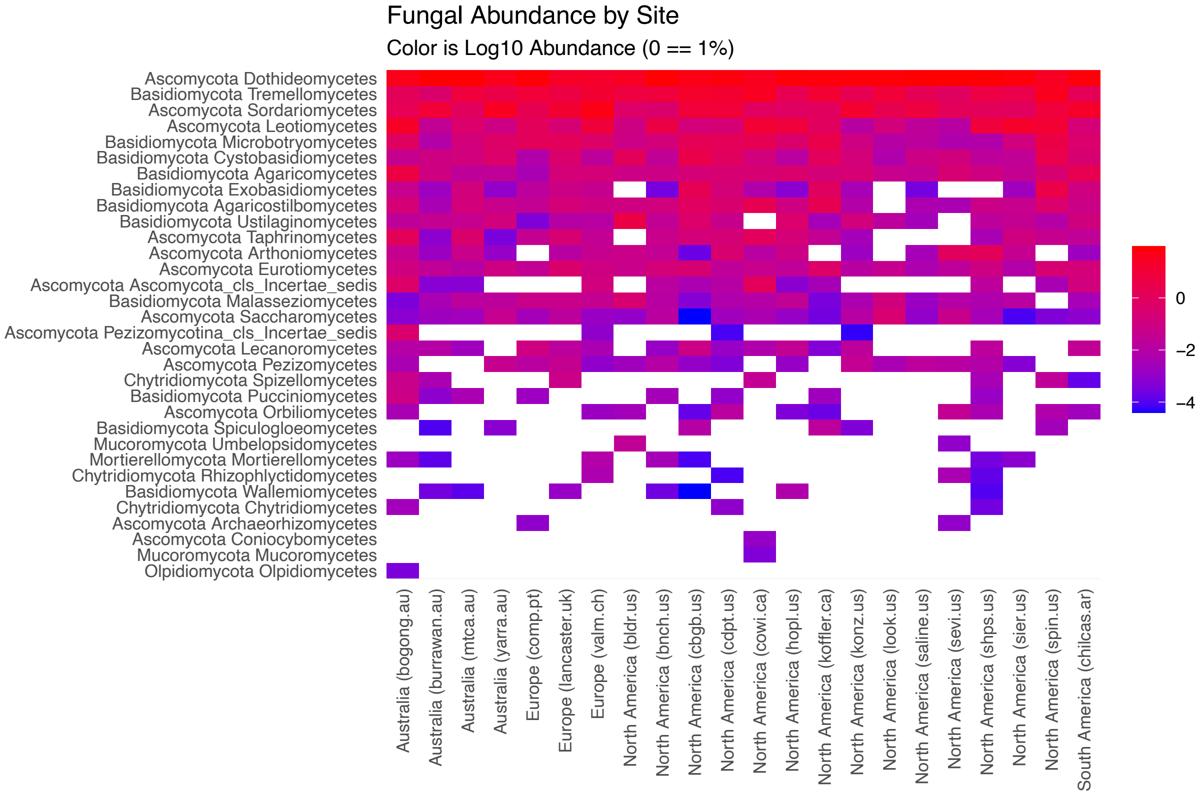

B.

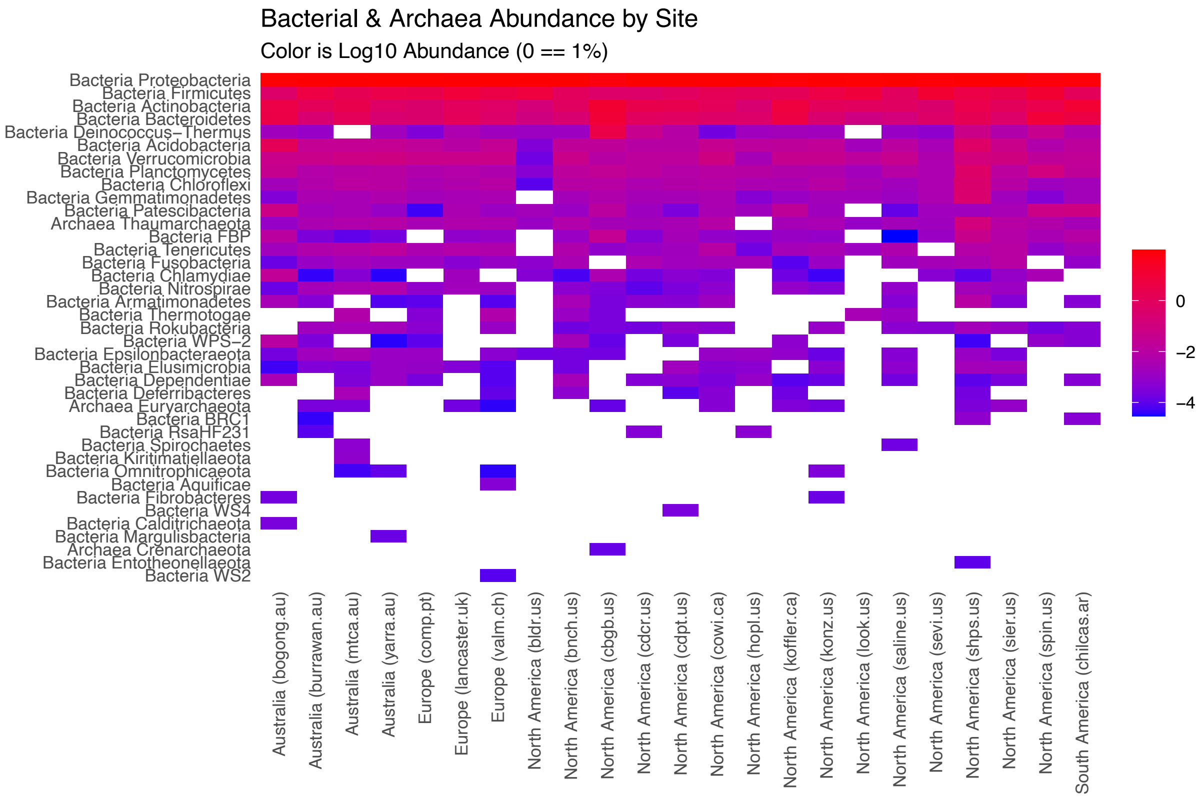
Bacterial & Archaea Abundance by Site
Color is Log10 Abundance (0 == 1%)
Bacteria Proteobacteria
Bacteria Firmicutes
Bacteria Actinobacteria
Bacteria Bacteroidetes
Bacteria Deinococcus−Thermus
Bacteria Acidobacteria
Bacteria Verrucomicrobia
Bacteria Planctomycetes
Bacteria Chloroflexi
Bacteria Gemmatimonadetes
Bacteria Patescibacteria
Archaea Thaumarchaeota
Bacteria FBP
Bacteria Tenericutes
Bacteria Fusobacteria
Bacteria Chlamydiae
Bacteria Nitrospirae
Bacteria Armatimonadetes
Bacteria Thermotogae
Bacteria Rokubacteria
Bacteria WPS−2
Bacteria Epsilonbacteraeota
Bacteria Elusimicrobia
Bacteria Dependentiae
Bacteria Deferribacteres
Archaea Euryarchaeota
Bacteria BRC1
Bacteria RsaHF231
Bacteria Spirochaetes
Bacteria Kiritimatiellaeota
Bacteria Omnitrophicaeota
Bacteria Aquificae
Bacteria Fibrobacteres
Bacteria WS4
Bacteria Calditrichaeota
Bacteria Margulisbacteria
Archaea Crenarchaeota
Bacteria Entotheonellaeota
Bacteria WS2
Australia (bogong.au)
Australia (burrawan.au)
Australia (mtca.au)
Australia (yarra.au)
Europe (comp.pt)
Europe (lancaster.uk)
Europe (valm.ch)
North America (bldr.us)
North America (bnch.us)
North America (cbgb.us)
North America (cdcr.us)
North America (cdpt.us)
North America (cowi.ca)
North America (hopl.us)
North America (koffler.ca)
North America (konz.us)
North America (look.us)
North America (saline.us)
North America (sevi.us)
North America (shps.us)
North America (sier.us)
North America (spin.us)
South America (chilcas.ar)
0
−2
−4

Table S1. Site characteristics, host species, and microbiome diversity.

Site Code	Continent	Country	Focal Host Species	Mean Annual Rainfall (mm yr^{-1})	Mean Annual Temperature (^{o}C)	Live Biomass (log g m^{-2})	Shade (Proportion)	Leaf-scale Fungal Diversity (ENS_{PIE})	Leaf-scale Prokaryote Diversity (ENS_{PIE})
bogong.au	Australia	Australia	*Poa hiemata*	1678	6.0	6.41	0.39	9.66	4.78
burrawan.au	Australia	Australia	*Eragrostis curvula*	643	18.2	5.88	0.63	9.93	2.32
mtca.au	Australia	Australia	*Austrostipa nitida*	324	17.7	6.02	0.65	6.00	2.08
yarra.au	Australia	Australia	*Setaria parviflora*	844	17.3	6.33	NA	4.77	1.62
valm.ch	Europe	Switzerland	*Festuca rubra*	681	0.1	5.52	NA	4.42	1.76
comp.pt	Europe	Portugal	*Avena barbata*	564	16.6	6.36	0.64	6.28	2.56
lancaster.uk	Europe	United Kingdom	*Holcus lanatus*	1522	8.0	5.40	0.39	4.03	2.23
cowi.ca	North America	Canada	*Aleopecurus pratensis*	762	10.4	6.17	0.92	8.17	2.37
koffler.ca	North America	Canada	*Bromus inermis*	853	6.3	6.58	0.93	16.61	11.51
bldr.us	North America	United States	*Sporobolus airoides*	487	9.9	5.02	NA	2.90	1.87
bnch.us	North America	United States	*Bromus carinatus*	1618	6.8	5.46	0.83	2.69	3.61
cbgb.us	North America	United States	*Schizachyrium scoparium*	871	9.3	6.68	0.91	25.33	24.93
cdcr.us	North America	United States	*Andropogon gerardii*	740	6.3	5.60	0.86	5.07	3.15
cdpt.us	North America	United States	*Stipa comata*	456	9.6	5.68	0.72	13.33	4.33
hopl.us	North America	United States	*Bromus hordeaceus*	1065	13.2	5.50	0.85	4.82	2.96
konz.us	North America	United States	*Andropogon gerardii*	889	12.1	6.70	NA	5.22	3.17
look.us	North America	United States	*Bromus carinatus*	1877	6.9	4.76	0.59	4.74	5.10
saline.us	North America	United States	*Andropogon gerardii*	608	12.1	5.80	0.58	6.26	2.47
sevi.us	North America	United States	*Bouteloua eriopoda*	252	13.1	4.98	NA	4.64	1.55
shps.us	North America	United States	*Pseudoroegneria spicata*	246	5.3	NA	NA	5.93	3.44
sier.us	North America	United States	*Bromus hordeaceus*	936	16.3	5.47	0.90	4.69	3.40
spin.us	North America	United States	*Poa pratensis*	1152	12.5	6.21	0.91	13.98	9.61
chilcas.ar	South America	Argentina	*Schenodorus phoenix*	955	15.1	6.70	0.85	10.01	11.60

Table S2. Drivers of population-scale fungal and prokaryotic (bacteria and archaea) diversity. Models are shown for the full set of 22 sites with all covariates, the subset of 17 sites at which we measured shading, and the subset of 14 sites at which we also measured soil chemistry. Results are based on multi-model inference from a suite of mixed effects models. The importance of the parameter is the sum of the Akaike weights summed across the set of models within 4 AICc units of the top model (lowest AICc) in which a parameter is included. Importance ranges from 0 (parameter has no explanatory weight) to 1 (parameter is in all top models). All tests are two-tailed.

Data Set	Response	Type of Averaging	Predictor	Estimate	Standard Error	z	p	Number of Models	Importance
**All Sites**	**Fungi**	**Full**	**Intercept**	**2.384**	**0.136**	**16.426**	**0.000**		
			**Plant Biomass**	**0.934**	**0.282**	**3.103**	**0.002**	**7.000**	**1.000**
			Plant Diversity	-0.062	0.208	0.288	0.773	2.000	0.188
			Host Abundance	-0.045	0.192	0.224	0.823	2.000	0.164
			Site Plant Richness	-0.007	0.092	0.074	0.941	1.000	0.098
			Precipitation	0.002	0.087	0.018	0.986	1.000	0.094
			Temperature	-0.001	0.089	0.011	0.991	1.000	0.094
		**Conditional**	**Intercept**	**2.384**	**0.136**	**16.426**	**0.000**		
			**Plant Biomass**	**0.934**	**0.282**	**3.103**	**0.002**	**7.000**	**1.000**
			Plant Diversity	-0.330	0.377	0.830	0.407	2.000	0.188
			Host Abundance	-0.272	0.405	0.640	0.522	2.000	0.164
			Site Plant Richness	-0.074	0.286	0.243	0.808	1.000	0.098
			Precipitation	0.018	0.284	0.059	0.953	1.000	0.094
			Temperature	-0.011	0.290	0.036	0.972	1.000	0.094
	**Prokaryotes**	**Full**	**Intercept**	**1.313**	**0.171**	**7.201**	**0.000**		
			Plant Biomass	0.490	0.468	1.019	0.308	17.000	0.661
			Temperature	-0.289	0.424	0.666	0.505	12.000	0.438
			Precipitation	0.192	0.338	0.552	0.581	12.000	0.360
			Plant Diversity	-0.077	0.333	0.227	0.820	10.000	0.312
			Host Abundance	-0.206	0.441	0.458	0.647	7.000	0.178
			Site Plant Richness	0.002	0.145	0.013	0.989	6.000	0.113
		**Conditional**	**Intercept**	**1.313**	**0.171**	**7.201**	**0.000**		
			Plant Biomass	0.742	0.381	1.837	0.066	17.000	0.661
			Temperature	-0.659	0.407	1.533	0.125	12.000	0.438
			Precipitation	0.532	0.368	1.358	0.175	12.000	0.360
			Plant Diversity	-0.436	0.684	0.617	0.537	10.000	0.312
			Host Abundance	-0.661	0.568	1.119	0.263	7.000	0.178
			Site Plant Richness	0.018	0.432	0.040	0.968	6.000	0.113

Data Set	Response	Type of Averaging	Predictor	Estimate	Standard Error	z	p	Number of Models	Importance
**Light Sites**	**Fungi**	**Full**	**Intercept**	**2.584**	**0.134**	**17.700**	**0.000**		
			**Plant Biomass**	**1.053**	**0.283**	**3.410**	**0.001**	**7.000**	**1.000**
			Temperature	-0.022	0.112	0.186	0.852	1.000	0.111
			Shading	-0.012	0.095	0.114	0.909	1.000	0.092
			Potential Evapotranspiration	-0.006	0.085	0.068	0.946	1.000	0.085
			Host Abundance	-0.003	0.090	0.026	0.979	1.000	0.082
			Precipitation	-0.000	0.087	0.004	0.997	1.000	0.082
			Site Plant Richness	-0.000	0.083	0.000	1.000	1.000	0.082
		**Conditional**	**Intercept**	**2.584**	**0.134**	**17.700**	**0.000**		
			**Plant Biomass**	**1.053**	**0.283**	**3.410**	**0.001**	**7.000**	**1.000**
			Temperature	-0.201	0.280	0.656	0.512	1.000	0.111
			Shading	-0.128	0.289	0.405	0.686	1.000	0.092
			Potential Evapotranspiration	-0.073	0.282	0.238	0.812	1.000	0.085
			Host Abundance	-0.032	0.314	0.092	0.927	1.000	0.082
			Precipitation	-0.004	0.303	0.012	0.990	1.000	0.082
			Site Plant Richness	-0.000	0.289	0.001	0.999	1.000	0.082
	**Prokaryotes**	**Full**	**Intercept**	**1.497**	**0.199**	**6.889**	**0.000**		
			Shading	0.347	0.462	0.726	0.468	9.000	0.478
			Temperature	-0.258	0.418	0.597	0.550	7.000	0.374
			Plant Biomass	0.209	0.390	0.517	0.605	7.000	0.328
			Precipitation	0.123	0.321	0.370	0.712	5.000	0.209
			Host Abundance	-0.009	0.136	0.062	0.951	3.000	0.068
			Site Plant Richness	0.001	0.118	0.011	0.991	3.000	0.066
		**Conditional**	**Intercept**	**1.497**	**0.199**	**6.889**	**0.000**		
			Shading	0.726	0.413	1.612	0.107	9.000	0.478
			Temperature	-0.690	0.411	1.537	0.124	7.000	0.374
			Plant Biomass	0.637	0.437	1.340	0.180	7.000	0.328
			Precipitation	0.588	0.467	1.164	0.244	5.000	0.209
			Host Abundance	-0.132	0.506	0.243	0.808	3.000	0.068
			Site Plant Richness	0.022	0.461	0.043	0.965	3.000	0.066

Data Set	Response	Type of Averaging	Predictor	Estimate	Standard Error	z	p	Number of Models	Importance
**Soil Sites**	**Fungi**	**Full**	**Intercept**	**2.681**	**0.156**	**15.331**	**0.000**		
			**Plant Biomass**	**1.069**	**0.339**	**2.825**	**0.005**	**10.000**	**1.000**
			Soil P	0.093	0.232	0.383	0.702	1.000	0.194
			Soil N	-0.020	0.118	0.154	0.878	1.000	0.070
			Soil C:N	-0.013	0.105	0.113	0.910	1.000	0.059
			Host Abundance	-0.012	0.099	0.107	0.914	1.000	0.058
			Shading	-0.010	0.091	0.096	0.924	1.000	0.056
			Precipitation	-0.009	0.093	0.086	0.931	1.000	0.054
			Temperature	-0.006	0.081	0.064	0.949	1.000	0.051
			Site Plant Richness	0.005	0.082	0.053	0.958	1.000	0.050
			Potential Evapotranspiration	0.004	0.079	0.051	0.960	1.000	0.050
		**Conditional**	**Intercept**	**2.681**	**0.156**	**15.331**	**0.000**		
			**Plant Biomass**	**1.069**	**0.339**	**2.825**	**0.005**	**10.000**	**1.000**
			Soil P	0.478	0.305	1.393	0.164	1.000	0.194
			Soil N	-0.281	0.356	0.702	0.482	1.000	0.070
			Soil C:N	-0.219	0.375	0.520	0.603	1.000	0.059
			Host Abundance	-0.203	0.364	0.496	0.620	1.000	0.058
			Shading	-0.173	0.349	0.442	0.658	1.000	0.056
			Precipitation	-0.165	0.368	0.399	0.690	1.000	0.054
			Temperature	-0.113	0.342	0.293	0.769	1.000	0.051
			Site Plant Richness	0.096	0.353	0.243	0.808	1.000	0.050
			Potential Evapotranspiration	0.089	0.342	0.233	0.816	1.000	0.050
	**Prokaryotes**	**Full**	**Intercept**	**1.623**	**0.179**	**8.087**	**0.000**		
			**Soil C:N**	**-1.168**	**0.490**	**2.217**	**0.027**	**7.000**	**0.952**
			Temperature	-0.359	0.448	0.769	0.442	3.000	0.480
			Soil P	0.184	0.367	0.482	0.630	3.000	0.283
			Site Plant Richness	-0.028	0.154	0.172	0.864	1.000	0.059
			Precipitation	0.026	0.143	0.169	0.865	1.000	0.058
			Shading	-0.026	0.162	0.149	0.882	1.000	0.051
		**Conditional**	**Intercept**	**1.623**	**0.179**	**8.087**	**0.000**		
			**Soil C:N**	**-1.227**	**0.424**	**2.618**	**0.009**	**7.000**	**0.952**
			Temperature	-0.748	0.358	1.858	0.063	3.000	0.480
			Soil P	0.651	0.415	1.414	0.157	3.000	0.283
			Site Plant Richness	-0.479	0.436	0.978	0.328	1.000	0.059
			Precipitation	0.445	0.409	0.968	0.333	1.000	0.058
			Shading	-0.510	0.522	0.859	0.390	1.000	0.051

Table S3. Drivers of leaf-scale fungal and prokaryotic (bacteria and archaea) diversity. Models are shown for the full set of 22 sites with all covariates, the subset of 17 sites at which we measured shading, and the subset of 14 sites at which we also measured soil chemistry. Results are based on multi-model inference from a suite of mixed effects models. The importance of the parameter is the sum of the Akaike weights summed across the set of models within 4 AICc units of the top model (lowest AICc) in which a parameter is included. Importance ranges from 0 (parameter has no explanatory weight) to 1 (parameter is in all top models). All tests are two tailed.

Data Set	Response	Type of Averaging	Predictor	Estimate	Standard Error	z	p	Number of Models	Importance
**All Sites**	**Fungi**	**Full**	**Intercept**	**1.789**	**0.054**	**32.695**	**0.000**		
			**Pop. Fungal Diversity**	**1.134**	**0.109**	**10.292**	**0.000**	**7.000**	**1.000**
			Temperature	-0.007	0.048	0.151	0.880	2.000	0.174
			Plant Diversity	0.006	0.039	0.144	0.885	2.000	0.173
			Host Abundance	-0.003	0.030	0.090	0.928	1.000	0.123
			Plant Biomass	0.002	0.031	0.052	0.958	1.000	0.120
			Precipitation	0.000	0.036	0.001	0.999	1.000	0.118
		**Conditional**	**Intercept**	**1.789**	**0.054**	**32.695**	**0.000**		
			**Pop. Fungal Diversity**	**1.134**	**0.109**	**10.292**	**0.000**	**7.000**	**1.000**
			Temperature	-0.042	0.108	0.382	0.702	2.000	0.174
			Plant Diversity	0.033	0.090	0.366	0.714	2.000	0.173
			Host Abundance	-0.022	0.082	0.265	0.791	1.000	0.123
			Plant Biomass	0.014	0.089	0.152	0.879	1.000	0.120
			Precipitation	0.000	0.104	0.002	0.998	1.000	0.118
	**Prokaryotes**	**Full**	**Intercept**	**1.149**	**0.051**	**22.335**	**0.000**		
			**Pop. Prokaryotic Diversity**	**1.293**	**0.114**	**11.260**	**0.000**	**11.000**	**1.000**
			Plant Diversity	-0.078	0.103	0.756	0.450	11.000	1.000
			Host Abundance	-0.159	0.110	1.434	0.151	7.000	0.812
			Plant Biomass	0.291	0.090	3.210	0.001	5.000	0.516
			Precipitation	-0.020	0.064	0.319	0.750	4.000	0.261
			Temperature	0.019	0.064	0.290	0.772	4.000	0.254
		**Conditional**	**Intercept**	**1.149**	**0.051**	**22.335**	**0.000**		
			**Pop. Prokaryotic Diversity**	**1.293**	**0.114**	**11.260**	**0.000**	**11.000**	**1.000**
			Plant Diversity	-0.152	0.098	1.545	0.122	11.000	1.000
			Host Abundance	-0.195	0.088	2.201	0.028	7.000	0.812
			Plant Biomass	0.291	0.090	3.210	0.001	5.000	0.516
			Precipitation	-0.078	0.105	0.740	0.459	4.000	0.261
			Temperature	0.074	0.110	0.663	0.507	4.000	0.254

Data Set	Response	Type of Averaging	Predictor	Estimate	Standard Error	z	p	Number of Models	Importance
**Light Sites**	**Fungi**	**Full**	**Intercept**	**1.916**	**0.063**	**30.157**	**0.000**		
			**Pop. Fungal Diversity**	**1.153**	**0.128**	**8.964**	**0.000**	**16.000**	**1.000**
			Shading	0.026	0.069	0.370	0.712	6.000	0.280
			Plant Diversity	0.014	0.052	0.274	0.784	6.000	0.246
			Plant Biomass	-0.008	0.043	0.179	0.858	3.000	0.155
			Host Abundance	-0.002	0.033	0.059	0.953	3.000	0.138
			Precipitation	0.003	0.046	0.069	0.945	3.000	0.137
			Temperature	0.001	0.047	0.021	0.983	3.000	0.136
		**Conditional**	**Intercept**	**1.916**	**0.063**	**30.157**	**0.000**		
			**Pop. Fungal Diversity**	**1.153**	**0.128**	**8.964**	**0.000**	**16.000**	**1.000**
			Shading	0.092	0.104	0.869	0.385	6.000	0.280
			Plant Diversity	0.058	0.092	0.630	0.529	6.000	0.246
			Plant Biomass	-0.051	0.100	0.500	0.617	3.000	0.155
			Host Abundance	-0.014	0.088	0.161	0.872	3.000	0.138
			Precipitation	0.023	0.123	0.190	0.849	3.000	0.137
			Temperature	0.007	0.129	0.057	0.955	3.000	0.136
	**Prokaryotes**	**Full**	**Intercept**	**1.305**	**0.060**	**21.545**	**0.000**		
			**Pop. Prokaryotic Diversity**	**1.326**	**0.132**	**9.999**	**0.000**	**11.000**	**1.000**
			**Host Abundance**	**-0.231**	**0.108**	**2.133**	**0.033**	**11.000**	**1.000**
			**Plant Biomass**	**0.418**	**0.105**	**3.947**	**0.000**	**10.000**	**0.956**
			Plant Diversity	-0.043	0.084	0.512	0.609	4.000	0.354
			Temperature	0.022	0.079	0.278	0.781	4.000	0.244
			Shading	0.009	0.055	0.165	0.869	3.000	0.187
			Precipitation	0.005	0.055	0.090	0.928	3.000	0.179
		**Conditional**	**Intercept**	**1.305**	**0.060**	**21.545**	**0.000**		
			**Pop. Prokaryotic Diversity**	**1.326**	**0.132**	**9.999**	**0.000**	**11.000**	**1.000**
			**Host Abundance**	**-0.242**	**0.098**	**2.451**	**0.014**	**11.000**	**1.000**
			**Plant Biomass**	**0.418**	**0.105**	**3.947**	**0.000**	**10.000**	**0.956**
			Plant Diversity	-0.122	0.102	1.188	0.235	4.000	0.354
			Temperature	0.091	0.140	0.646	0.518	4.000	0.244
			Shading	0.049	0.120	0.406	0.685	3.000	0.187
			Precipitation	0.028	0.128	0.217	0.828	3.000	0.179

Data Set	Response	Type of Averaging	Predictor	Estimate	Standard Error	z	p	Number of Models	Importance
**Soil Sites**	**Fungi**	**Full**	**Intercept**	**2.001**	**0.065**	**30.506**	**0.000**		
			**Pop. Fungal Diversity**	**1.211**	**0.142**	**8.428**	**0.000**	**78.000**	**1.000**
			Soil C:N	-0.050	0.094	0.527	0.598	46.000	0.560
			Shading	0.124	0.149	0.825	0.410	27.000	0.343
			Soil N	0.054	0.114	0.472	0.637	32.000	0.339
			Host Abundance	0.032	0.077	0.414	0.679	24.000	0.253
			Plant Biomass	-0.026	0.076	0.334	0.738	21.000	0.224
			Soil P	0.009	0.051	0.167	0.867	16.000	0.145
			Precipitation	0.003	0.065	0.045	0.965	14.000	0.133
			Plant Diversity	-0.003	0.037	0.084	0.933	12.000	0.123
			Temperature	0.007	0.054	0.131	0.896	11.000	0.107
		**Conditional**	**Intercept**	**2.001**	**0.065**	**30.506**	**0.000**		
			**Pop. Fungal Diversity**	**1.211**	**0.142**	**8.428**	**0.000**	**78.000**	**1.000**
			Soil C:N	-0.146	0.110	1.314	0.189	46.000	0.560
			Shading	0.221	0.136	1.614	0.107	27.000	0.343
			Soil N	0.160	0.147	1.080	0.280	32.000	0.339
			Host Abundance	0.127	0.108	1.170	0.242	24.000	0.253
			Plant Biomass	-0.114	0.125	0.904	0.366	21.000	0.224
			Soil P	0.069	0.129	0.532	0.595	16.000	0.145
			Precipitation	0.022	0.176	0.123	0.902	14.000	0.133
			Plant Diversity	-0.029	0.110	0.265	0.791	12.000	0.123
			Temperature	0.049	0.135	0.363	0.717	11.000	0.107
	**Prokaryotes**	**Full**	**Intercept**	**1.445**	**0.073**	**19.570**	**0.000**		
			**Pop. Prokaryotic Diversity**	**1.286**	**0.165**	**7.721**	**0.000**	**26.000**	**1.000**
			**Host Abundance**	**-0.282**	**0.142**	**1.976**	**0.048**	**26.000**	**1.000**
			Plant Biomass	0.496	0.124	3.963	0.000	24.000	0.943
			Plant Diversity	-0.071	0.114	0.617	0.537	12.000	0.422
			Temperature	0.065	0.126	0.515	0.606	13.000	0.373
			Soil C:N	-0.006	0.049	0.124	0.901	4.000	0.124
			Soil N	-0.004	0.051	0.071	0.943	4.000	0.120
			Precipitation	-0.003	0.054	0.047	0.962	4.000	0.118
			Soil P	-0.005	0.055	0.084	0.933	4.000	0.117
			Shading	-0.000	0.052	0.004	0.997	4.000	0.113
		**Conditional**	**Intercept**	**1.445**	**0.073**	**19.570**	**0.000**		
			**Pop. Prokaryotic Diversity**	**1.286**	**0.165**	**7.721**	**0.000**	**26.000**	**1.000**
			**Host Abundance**	**-0.299**	**0.127**	**2.325**	**0.020**	**26.000**	**1.000**
			**Plant Biomass**	**0.496**	**0.124**	**3.963**	**0.000**	**24.000**	**0.943**
			Plant Diversity	-0.168	0.121	1.372	0.170	12.000	0.422
			Temperature	0.175	0.153	1.135	0.257	13.000	0.373
			Soil C:N	-0.049	0.131	0.375	0.708	4.000	0.124
			Soil N	-0.031	0.147	0.211	0.833	4.000	0.120
			Precipitation	-0.022	0.156	0.138	0.890	4.000	0.118
			Soil P	-0.040	0.156	0.252	0.801	4.000	0.117
			Shading	-0.002	0.155	0.011	0.991	4.000	0.113

Table S4. Nutrient and herbivore effects on leaf-scale fungal and prokaryotic (bacteria and archaea) diversity. Results are based on mixed-effects models, and all tests are two tailed.

	Fungal Diversity			Prokaryotic Diversity		
**Predictors**	**Estimates**	**Standard Error**	**p**	**Estimates**	**Standard Error**	**p**
**Intercept**	**1.841**	**0.154**	**<0.001**	**1.220**	**0.187**	**<0.001**
**Nutrient**	**0.136**	**0.069**	**0.048**	**0.144**	**0.065**	**0.028**
**Fence**	**0.164**	**0.071**	**0.021**	0.076	0.068	0.260
**Nutrient*Fence**	-0.180	0.101	0.076	-0.043	0.096	0.652
**Random Effects**						
σ^2	0.2169			0.1535		
τ_{00}	0.0370 $_{plot:(block:site)}$			0.0475 $_{plot:(block:site)}$		
	0.0049 $_{block:site}$			0.0867 $_{block:site}$		
	0.3411 $_{site}$			0.4991 $_{site}$		
Observations	515			515		

Table S5. Nutrient and herbivore effects on the host community. Results are based on mixed-effects models, and all tests are two tailed.

	Plant Biomass			Shading			Plant Diversity			Host Abundance		
**Predictors**	**Estimates**	**Standard Error**	**p**	**Estimates**	**Standard Error**	**p**	**Estimates**	**Standard Error**	**p**	**Estimates**	**Standard Error**	**p**
**Intercept**	**5.615**	**0.126**	**<0.001**	**0.667**	**0.048**	**<0.001**	**1.391**	**0.098**	**<0.001**	**-2.222**	**0.353**	**<0.001**
**Nutrient**	**0.469**	**0.051**	**<0.001**	**0.108**	**0.018**	**<0.001**	**-0.323**	**0.045**	**<0.001**	**-0.509**	**0.194**	**0.009**
**Fence**	0.063	0.053	0.241	0.010	0.019	0.609	**-0.107**	**0.046**	**0.021**	-0.146	0.201	0.467
**Nutrient*Fence**	-0.093	0.076	0.223	**0.083**	**0.027**	**0.002**	0.016	0.066	0.811	-0.109	0.286	0.704
**Random Effects**												
σ^2	0.1809			0.0228			0.1359			2.5683		
τ_{00}	0.1163 $_{block:site}$			0.0054 $_{block:site}$			0.0657 $_{block:site}$			0.6348 $_{block:site}$		
	0.1931 $_{site}$			0.0327 $_{site}$			0.1152 $_{site}$			1.4846 $_{site}$		
Observations	515			515			515			515		

Table S6. Structural Equation Models (SEMs) linking nutrient addition and herbivore exclusion to changes in the plant community and microbiome diversity. The full model (Model 1) does not differ from the model in which all nutrient and herbivore effects were mediated through changes in the plant community (Model 2; p=0.2875) or the most parsimonious model in which all effects are mediated through changes in shading (Model 3; p=0.181). Double-headed arrows indicate relationships modeled as correlated errors. All tests are two-tailed.

Model 1: Global goodness-of-fit: Fisher's C = 3.157; p= 0.206 on 2 DF; AIC = 95.157

Response		Predictor	Estimate	Standard Error	DF	p	Standard Estimate
**Host Abundance**	⇐	**Nutrient**	**-0.519**	**0.208**	**496.7**	**0.013**	**-0.121**
Host Abundance	⇐	Fence	-0.107	0.214	497.2	0.617	-0.025
Host Abundance	⇐	Nutrient:Fence	-0.115	0.307	496.6	0.708	-0.022
**Plant Biomass**	⇐	**Nutrient**	**0.463**	**0.061**	**496.5**	**0.000**	**0.330**
Plant Biomass	⇐	Fence	0.068	0.063	496.8	0.287	0.048
Plant Biomass	⇐	Nutrient:Fence	-0.081	0.091	496.4	0.371	-0.048
**Shading**	⇐	**Plant Biomass**	**0.134**	**0.013**	**507.5**	**0.000**	**0.375**
**Shading**	⇐	**Nutrient**	**0.044**	**0.019**	**497.5**	**0.020**	**0.088**
Shading	⇐	Fence	0.004	0.018	495.5	0.816	0.009
**Shading**	⇐	**Nutrient:Fence**	**0.097**	**0.026**	**495.3**	**0.000**	**0.162**
**Fungal Diversity**	⇐	**Nutrient**	**0.146**	**0.066**	**494.8**	**0.026**	**0.096**
**Fungal Diversity**	⇐	**Fence**	**0.172**	**0.063**	**492.6**	**0.006**	**0.112**
Fungal Diversity	⇐	Plant Biomass	-0.032	0.050	506.2	0.515	-0.030
**Fungal Diversity**	⇐	**Shading**	**0.380**	**0.151**	**507.0**	**0.013**	**0.125**
**Fungal Diversity**	⇐	**Plant Diversity**	**0.112**	**0.056**	**505.2**	**0.048**	**0.085**
Fungal Diversity	⇐	Host Abundance	-0.010	0.013	503.4	0.444	-0.029
**Fungal Diversity**	⇐	**Nutrient:Fence**	**-0.215**	**0.091**	**493.0**	**0.018**	**-0.118**
Prokaryotic Diversity	⇐	Nutrient	0.089	0.066	494.2	0.178	0.051
Prokaryotic Diversity	⇐	Fence	0.079	0.063	492.4	0.210	0.045
Prokaryotic Diversity	⇐	Plant Biomass	0.047	0.050	504.7	0.353	0.038
**Prokaryotic Diversity**	⇐	**Shading**	**0.384**	**0.153**	**506.3**	**0.013**	**0.111**
Prokaryotic Diversity	⇐	Plant Diversity	0.023	0.057	503.5	0.683	0.016
Prokaryotic Diversity	⇐	Host Abundance	-0.022	0.013	501.6	0.096	-0.055
Prokaryotic Diversity	⇐	Nutrient:Fence	-0.085	0.091	492.8	0.354	-0.041
**Plant Diversity**	⇐	**Shading**	**-0.463**	**0.111**	**504.6**	**0.000**	**-0.200**
**Plant Diversity**	⇐	**Nutrient**	**-0.276**	**0.051**	**498.5**	**0.000**	**-0.238**
Plant Diversity	⇐	Fence	-0.082	0.051	495.9	0.113	-0.070
Plant Diversity	⇐	Nutrient:Fence	0.045	0.074	495.8	0.545	0.032
**Fungal Diversity**	⇔	**Prokaryotic Diversity**	**0.208**	**-**	**515.0**	**0.000**	**0.208**
**Plant Diversity**	⇔	**Plant Biomass**	**-0.168**	**-**	**515.0**	**0.000**	**-0.168**
**Plant Diversity**	⇔	**Host Abundance**	**0.154**	**-**	**515.0**	**0.000**	**0.154**
**Host Abundance**	⇔	**Plant Biomass**	**0.102**	**-**	**515.0**	**0.010**	**0.102**

Response	Marginal r^2	Conditional r^2
Host Abundance	0.02	0.37
Plant Biomass	0.09	0.51
Shading	0.21	0.68
Fungal Diversity	0.02	0.60
Prokaryotic Diversity	0.02	0.66
Plant Diversity	0.12	0.49

Model 2: Global goodness-of-fit: Fisher's C = 12.847; p= 0.232 on 10 DF; AIC = 92.847

Response		Predictor	Estimate	Standard Error	DF	p	Standard Estimate
**Host Abundance**	⇐	**Nutrient**	**-0.519**	**0.208**	**496.7**	**0.013**	**-0.121**
Host Abundance	⇐	Fence	-0.107	0.214	497.2	0.617	-0.025
Host Abundance	⇐	Nutrient:Fence	-0.115	0.307	496.6	0.708	-0.022
**Plant Biomass**	⇐	**Nutrient**	**0.463**	**0.061**	**496.5**	**0.000**	**0.330**
Plant Biomass	⇐	Fence	0.068	0.063	496.8	0.287	0.048
Plant Biomass	⇐	Nutrient:Fence	-0.081	0.091	496.4	0.371	-0.048
**Shading**	⇐	**Plant Biomass**	**0.134**	**0.013**	**507.5**	**0.000**	**0.375**
**Shading**	⇐	**Nutrient**	**0.044**	**0.019**	**497.5**	**0.020**	**0.088**
Shading	⇐	Fence	0.004	0.018	495.5	0.816	0.009
**Shading**	⇐	**Nutrient:Fence**	**0.097**	**0.026**	**495.3**	**0.000**	**0.162**
**Plant Diversity**	⇐	**Shading**	**-0.463**	**0.111**	**504.6**	**0.000**	**-0.200**
**Plant Diversity**	⇐	**Nutrient**	**-0.276**	**0.051**	**498.5**	**0.000**	**-0.238**
Plant Diversity	⇐	Fence	-0.082	0.051	495.9	0.113	-0.070
Plant Diversity	⇐	Nutrient:Fence	0.045	0.074	495.8	0.545	0.032
Fungal Diversity	⇐	Plant Biomass	-0.016	0.049	509.4	0.751	-0.014
**Fungal Diversity**	⇐	**Shading**	**0.387**	**0.145**	**510.0**	**0.008**	**0.127**
Fungal Diversity	⇐	Plant Diversity	0.094	0.055	508.5	0.091	0.071
Fungal Diversity	⇐	Host Abundance	-0.012	0.013	507.1	0.364	-0.034
Prokaryotic Diversity	⇐	Plant Biomass	0.059	0.049	507.8	0.231	0.047
**Prokaryotic Diversity**	⇐	**Shading**	**0.413**	**0.145**	**508.9**	**0.005**	**0.119**
Prokaryotic Diversity	⇐	Plant Diversity	0.008	0.055	506.7	0.879	0.006
Prokaryotic Diversity	⇐	Host Abundance	-0.024	0.013	505.1	0.069	-0.060
**Fungal Diversity**	⇔	**Prokaryotic Diversity**	**0.215**	**-**	**515.0**	**0.000**	**0.215**
**Plant Diversity**	⇔	**Plant Biomass**	**-0.168**	**-**	**515.0**	**0.000**	**-0.168**
**Plant Diversity**	⇔	**Host Abundance**	**0.154**	**-**	**515.0**	**0.000**	**0.154**
**Host Abundance**	⇔	**Plant Biomass**	**0.102**	**-**	**515.0**	**0.010**	**0.102**

Response	Marginal r^2	Conditional r^2
Host Abundance	0.02	0.37
Plant Biomass	0.09	0.51
Shading	0.21	0.68
Fungal Diversity	0.12	0.49
Prokaryotic Diversity	0.02	0.59
Plant Diversity	0.02	0.65

Model 3: Fisher's C = 28.712; p = 0.153 on 22 DF; AIC = 96.712

Response		Predictor	Estimate	Standard Error	DF	p	Standard Estimate
**Host Abundance**	⇐	**Nutrient**	**-0.519**	**0.208**	**496.7**	**0.013**	**-0.121**
Host Abundance	⇐	Fence	-0.107	0.214	497.2	0.617	-0.025
Host Abundance	⇐	Nutrient:Fence	-0.115	0.307	496.6	0.708	-0.022
**Plant Biomass**	⇐	**Nutrient**	**0.463**	**0.061**	**496.5**	**0.000**	**0.330**
Plant Biomass	⇐	Fence	0.068	0.063	496.8	0.287	0.048
Plant Biomass	⇐	Nutrient:Fence	-0.081	0.091	496.4	0.371	-0.048
**Shading**	⇐	**Plant Biomass**	**0.134**	**0.013**	**507.5**	**0.000**	**0.375**
**Shading**	⇐	**Nutrient**	**0.044**	**0.019**	**497.5**	**0.020**	**0.088**
Shading	⇐	Fence	0.004	0.018	495.5	0.816	0.009
**Shading**	⇐	**Nutrient:Fence**	**0.097**	**0.026**	**495.3**	**0.000**	**0.162**
**Plant Diversity**	⇐	**Shading**	**-0.463**	**0.111**	**504.6**	**0.000**	**-0.200**
**Plant Diversity**	⇐	**Nutrient**	**-0.276**	**0.051**	**498.5**	**0.000**	**-0.238**
Plant Diversity	⇐	Fence	-0.082	0.051	495.9	0.113	-0.070
Plant Diversity	⇐	Nutrient:Fence	0.045	0.074	495.8	0.545	0.032
**Fungal Diversity**	⇐	**Shading**	**0.302**	**0.123**	**512.1**	**0.015**	**0.099**
**Prokaryotic Diversity**	⇐	**Shading**	**0.514**	**0.124**	**509.7**	**0.000**	**0.148**
**Fungal Diversity**	⇔	**Prokaryotic Diversity**	**0.214**	**-**	**515.0**	**0.000**	**0.214**
**Plant Diversity**	⇔	**Plant Biomass**	**-0.168**	**-**	**515.0**	**0.000**	**-0.168**
**Plant Diversity**	⇔	**Host Abundance**	**0.154**	**-**	**515.0**	**0.000**	**0.154**
**Host Abundance**	⇔	**Plant Biomass**	**0.102**	**-**	**515.0**	**0.010**	**0.102**

Response	Marginal r^2	Conditional r^2
Host Abundance	0.02	0.37
Plant Biomass	0.09	0.51
Shading	0.21	0.68
Fungal Diversity	0.12	0.49
Prokaryotic Diversity	0.01	0.57
Plant Diversity	0.02	0.66
