# Supplementary material for: Globally consistent response of plant microbiome diversity across hosts and continents to soil nutrients and herbivores
Source: Nat Commun. 2023 Jun 14;14:3516. doi: 10.1038/s41467-023-39179-w (PMC10267103; doi:10.1038/s41467-023-39179-w)
Supplement: Supplementary file 2 — Reporting Summary [file 41467_2023_39179_MOESM2_ESM.pdf]

nature portfolio

Corresponding author(s): Eric Seabloom

Last updated by author(s): Mar 17, 2023

Reporting Summary

Nature Portfolio wishes to improve the reproducibility of the work that we publish. This form provides structure for consistency and transparency in reporting. For further information on Nature Portfolio policies, see our Editorial Policies and the Editorial Policy Checklist.

Statistics

For all statistical analyses, confirm that the following items are present in the figure legend, table legend, main text, or Methods section.

n/a	Confirmed	
☐	☒	The exact sample size (*n*) for each experimental group/condition, given as a discrete number and unit of measurement
☐	☒	A statement on whether measurements were taken from distinct samples or whether the same sample was measured repeatedly
☐	☒	The statistical test(s) used AND whether they are one- or two-sided<br>*Only common tests should be described solely by name; describe more complex techniques in the Methods section.*
☐	☒	A description of all covariates tested
☐	☒	A description of any assumptions or corrections, such as tests of normality and adjustment for multiple comparisons
☐	☒	A full description of the statistical parameters including central tendency (e.g. means) or other basic estimates (e.g. regression coefficient) AND variation (e.g. standard deviation) or associated estimates of uncertainty (e.g. confidence intervals)
☐	☒	For null hypothesis testing, the test statistic (e.g. F, t, r) with confidence intervals, effect sizes, degrees of freedom and P value noted<br>*Give P values as exact values whenever suitable.*
☒	☐	For Bayesian analysis, information on the choice of priors and Markov chain Monte Carlo settings
☒	☐	For hierarchical and complex designs, identification of the appropriate level for tests and full reporting of outcomes
☒	☐	Estimates of effect sizes (e.g. Cohen's *d*, Pearson's *r*), indicating how they were calculated

Our web collection on statistics for biologists contains articles on many of the points above.

Software and code

Policy information about availability of computer code

Data collection	No software was used for data collection.
Data analysis	All analyses were conducted using R version 4.1.2. Specific R libraries with version numbers are listed in the Methods.

For manuscripts utilizing custom algorithms or software that are central to the research but not yet described in published literature, software must be made available to editors and reviewers. We strongly encourage code deposition in a community repository (e.g. GitHub). See the Nature Portfolio guidelines for submitting code & software for further information.

Data

Policy information about availability of data

All manuscripts must include a data availability statement. This statement should provide the following information, where applicable:

- Accession codes, unique identifiers, or web links for publicly available datasets
- A description of any restrictions on data availability
- For clinical datasets or third party data, please ensure that the statement adheres to our policy

All data used in this manuscript will be archived at the Environmental Data Initiative (https://environmentaldatainitiative.org/). The data DOI will be included at the end of the article upon acceptance.

Human research participants

Policy information about studies involving human research participants and Sex and Gender in Research.

Reporting on sex and gender	NA
Population characteristics	NA
Recruitment	NA
Ethics oversight	NA

Note that full information on the approval of the study protocol must also be provided in the manuscript.

Field-specific reporting

Please select the one below that is the best fit for your research. If you are not sure, read the appropriate sections before making your selection.

☐ Life sciences ☐ Behavioural & social sciences ☒ Ecological, evolutionary & environmental sciences

For a reference copy of the document with all sections, see nature.com/documents/nr-reporting-summary-flat.pdf

Ecological, evolutionary & environmental sciences study design

All studies must disclose on these points even when the disclosure is negative.

Study description	We conducted this work using sites that are part of the Nutrient Network Experiment (NutNet; www.nutnet.org), a globally replicated experiment manipulating elemental nutrient supplies and herbivore density in grasslands worldwide. Data were collected within the context of an experiment composed of a factorial combination of two treatments: Nutrient Addition and Herbivore reduction applied to 5 x 5 m plots.
Research sample	We used amplicon sequencing to measure relative abundances of fungal (ITS1) and prokaryotic (16S) diversity in the leaves of the most widespread grass at each of 23 grassland sites. Research sites were selected, because they were part of the Nutrient Network experiment and had been implementing the required treatments.
Sampling strategy	At peak biomass, we collected the most mature, non-senescent leaves totaling at least 250 mg of fresh tissue from each of three individuals of the focal grass species in each plot. Sample size was determined to provide sufficient tissue for DNA extraction.
Data collection	Amplicon sequencing was performed for the v4 16S rRNA and ITS-1 regions using 2 x 250 paired end on an Illumina MiSeq platform, according to standard protocols at the University of Minnesota Genomics Center (UMGC). Data were recorded directly by UMGC staff.
Timing and spatial scale	Data were collected within 5x5 m plots at 23 grasslands sites in seven countries on four continents. Data were collected at the same time and in the same plots to ensure they were directly comparable.
Data exclusions	All exclusion criteria were established before the analyses were conducted. We removed sequences that were identified as cyanobacteria, because these were likely to be chloroplasts. We removed sequences that were non-fungal eukaryotes, because fungi were the only eukaryotes were studying. We also removed samples with less than 1000 reads (5% of total samples), as these did not include enough sequences to represent the community.
Reproducibility	We replicated the experiment at 23 grasslands sites in seven countries on four continents.
Randomization	We used a completely randomized block design.
Blinding	NA

Did the study involve field work? ☒ Yes ☐ No

Field work, collection and transport

Field conditions	At the same time as we collected samples for DNA sequencing, we measured physical and biological characteristics of the environment that we expected could control microbiome diversity based on the theoretical and empirical evidence discussed above (plant biomass, plant diversity, host abundance, shading, soil resources, and climate). Specifically, we examined the effects of potential drivers of microbiome diversity including climate (MAP, MAT, and MAP divided by potential evapotranspiration),

	aboveground plant biomass, plant diversity, shading, and soil chemistry (soil N, P, C:N, and pH) (Tables S2 & S3). Details are presented in the manuscript and supplemental material.
Location	We replicated the experiment at 23 grasslands sites in seven countries on four continents (Table S1). these sites span globally relevant gradients in elevation (15 - 2320 m), latitude (37o S - 54o N), mean annual precipitation (MAP; 246 - 1877 mm yr-1), mean annual temperature (MAT: 0 - 18 oC), soil nutrients (0.03 – 1.3 % nitrogen, N, 13 – 234 ppm phosphorus, P), aboveground live biomass (117 - 813 g m-2), and plant richness (3 - 22 species m-2, 11 - 86 species site-1).
Access & import/export	All sample materials were shipped with appropriate import permits. Local site coordinators at each site were responsible for ensuring that sampling requirements for each sites were met.
Disturbance	The plots were not disturbed.

Reporting for specific materials, systems and methods

We require information from authors about some types of materials, experimental systems and methods used in many studies. Here, indicate whether each material, system or method listed is relevant to your study. If you are not sure if a list item applies to your research, read the appropriate section before selecting a response.

Materials & experimental systems

n/a	Involved in the study	
☒	☐	Antibodies
☒	☐	Eukaryotic cell lines
☒	☐	Palaeontology and archaeology
☒	☐	Animals and other organisms
☒	☐	Clinical data
☒	☐	Dual use research of concern

Methods

n/a	Involved in the study	
☒	☐	ChIP-seq
☒	☐	Flow cytometry
☒	☐	MRI-based neuroimaging
